# Supplementary material for: BetaII-spectrin gaps and patches emerge from the patterned assembly of the actin/spectrin membrane skeleton in human motor neuron axons
Source: eLife. 2026 Sep 8;14:RP108021. doi: 10.7554/eLife.108021 (PMC13553066; doi:10.7554/eLife.108021)

FIGURE 4 - SOURCE DATA 1

1 FIGURE 4 A

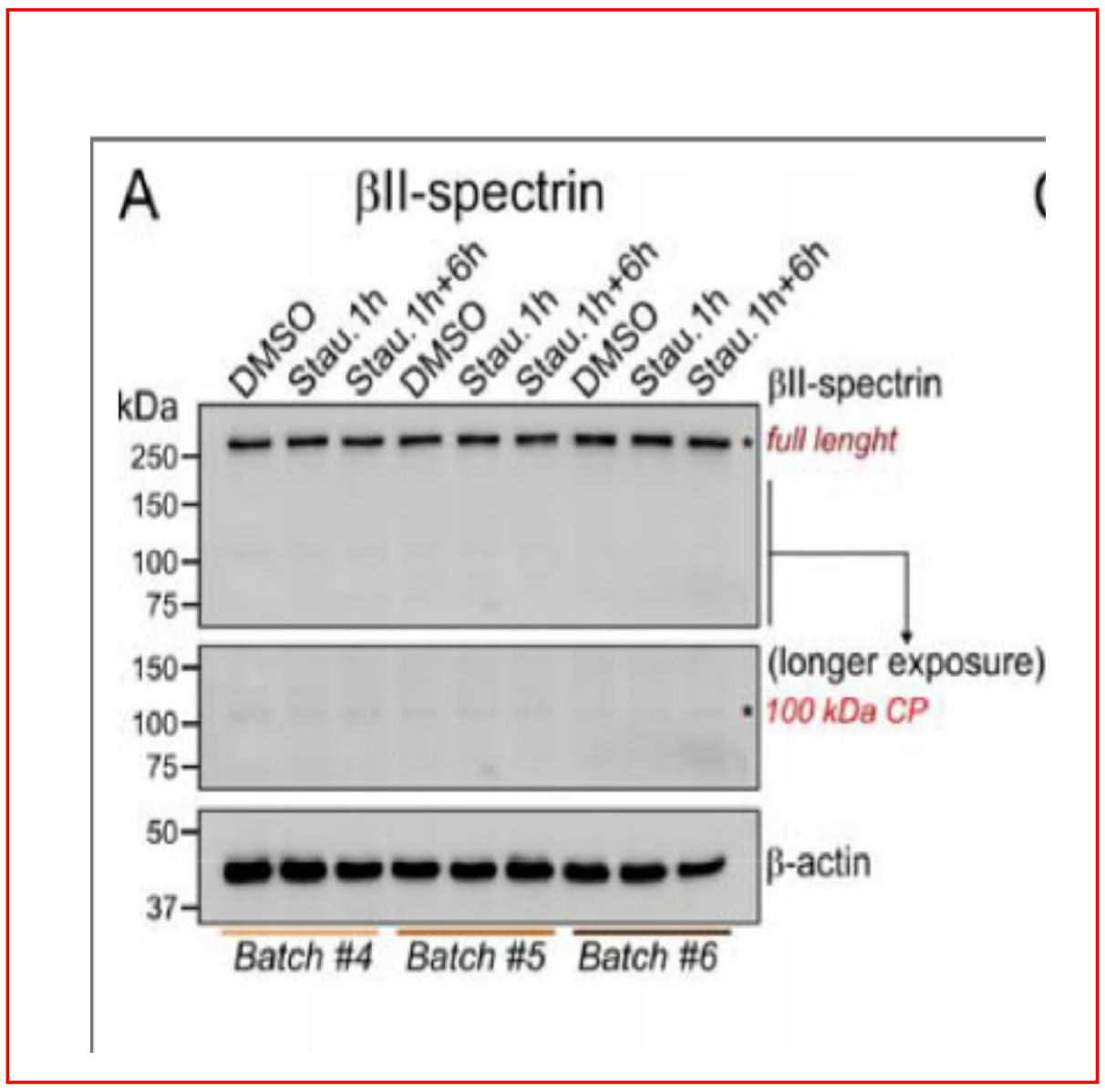

2

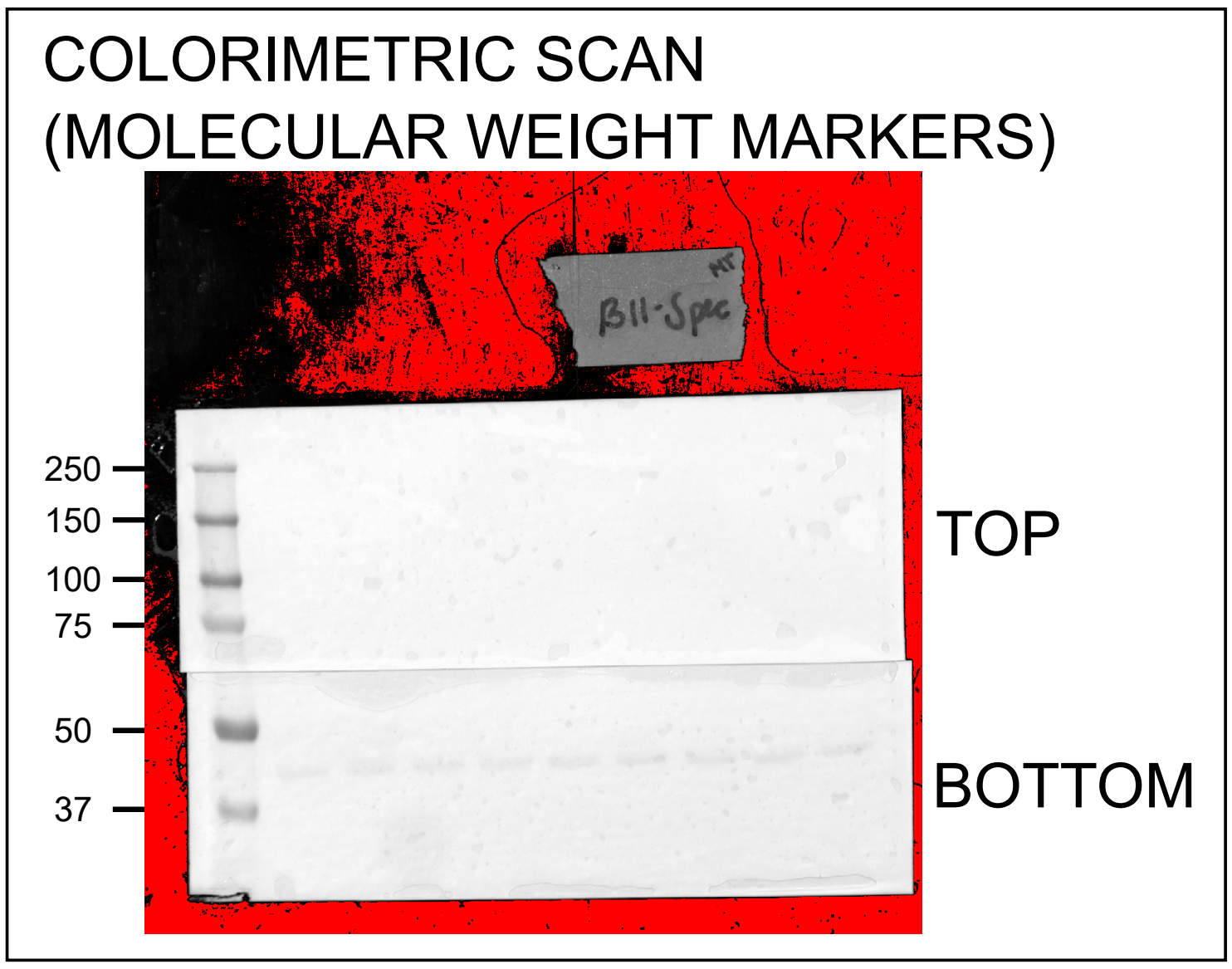

3

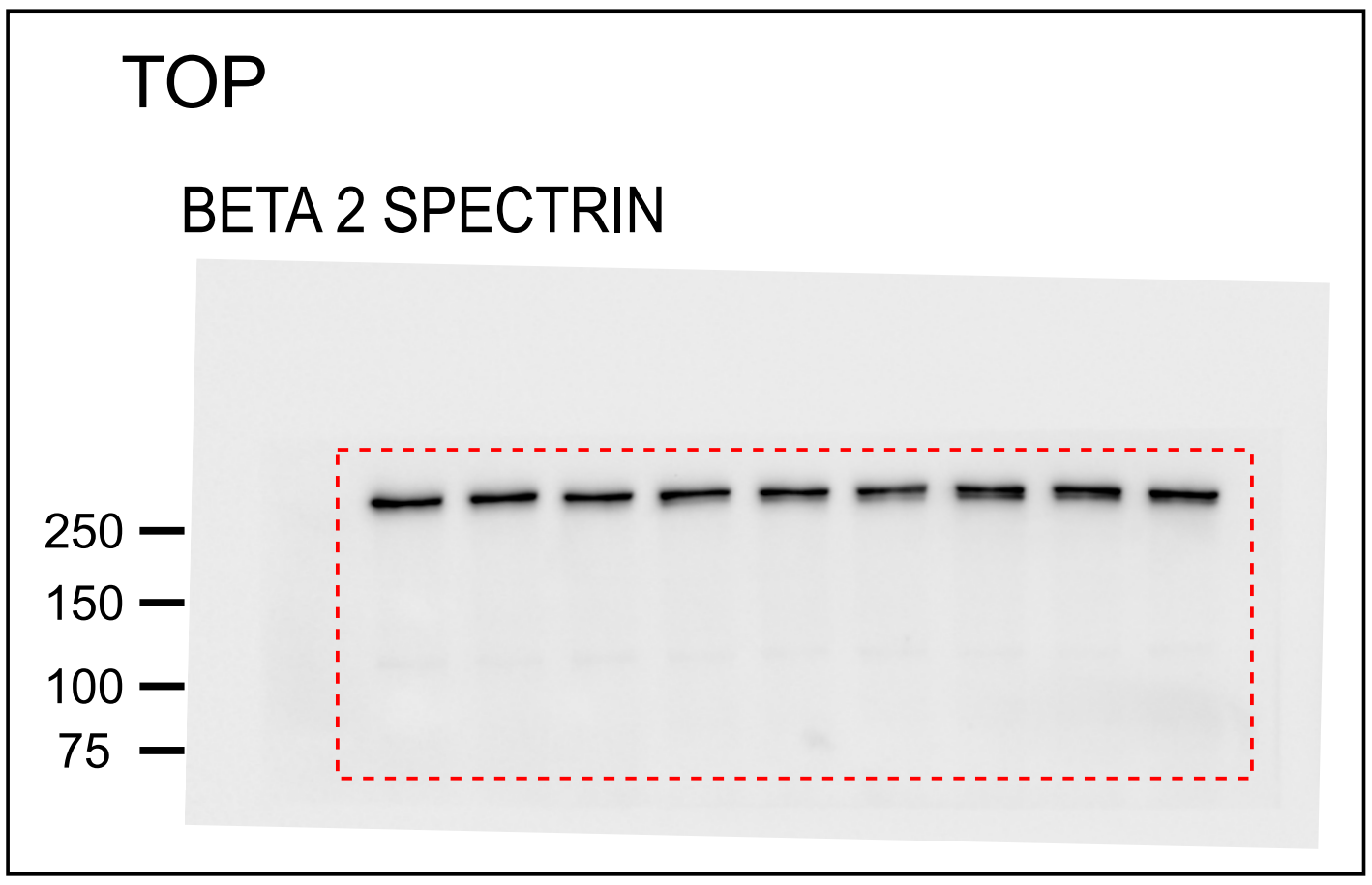

4

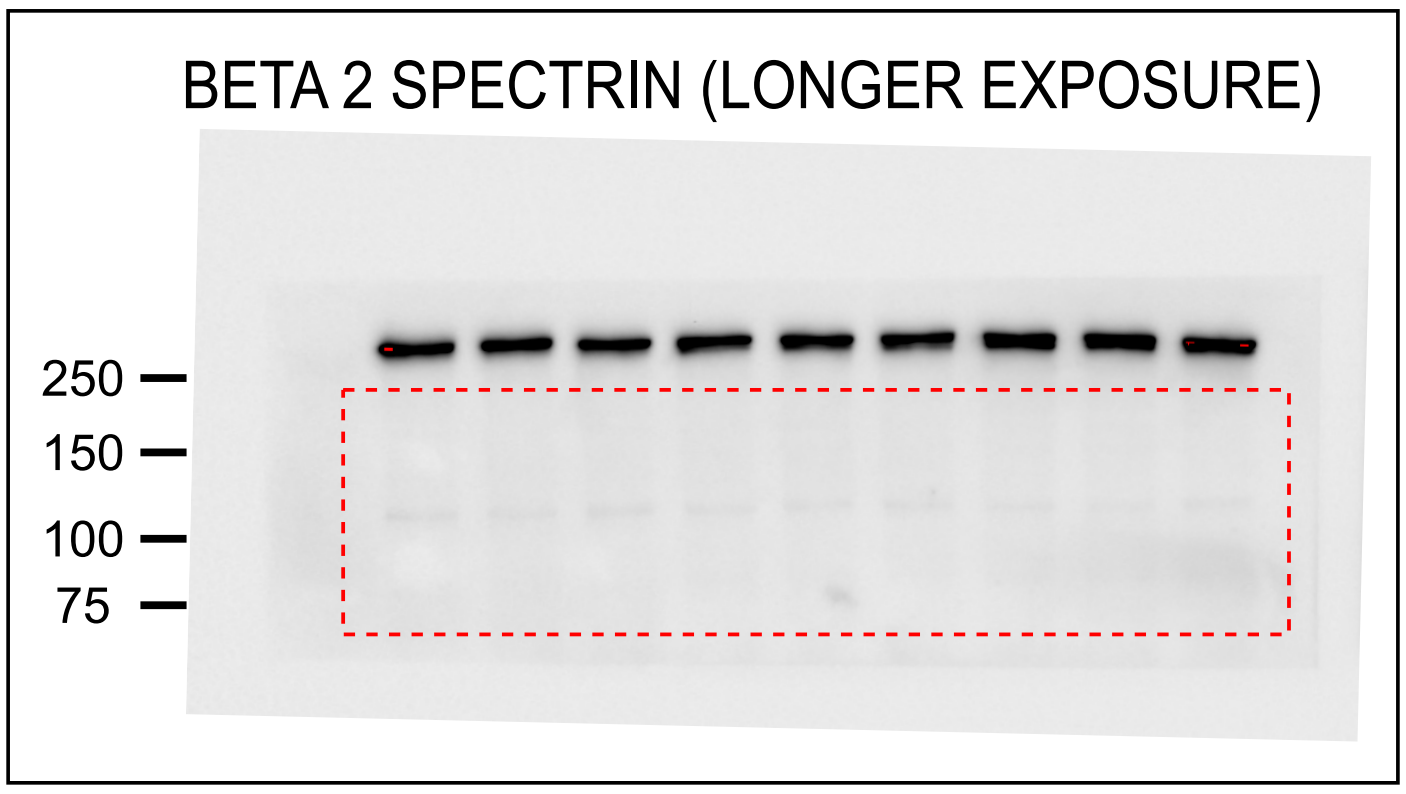

5

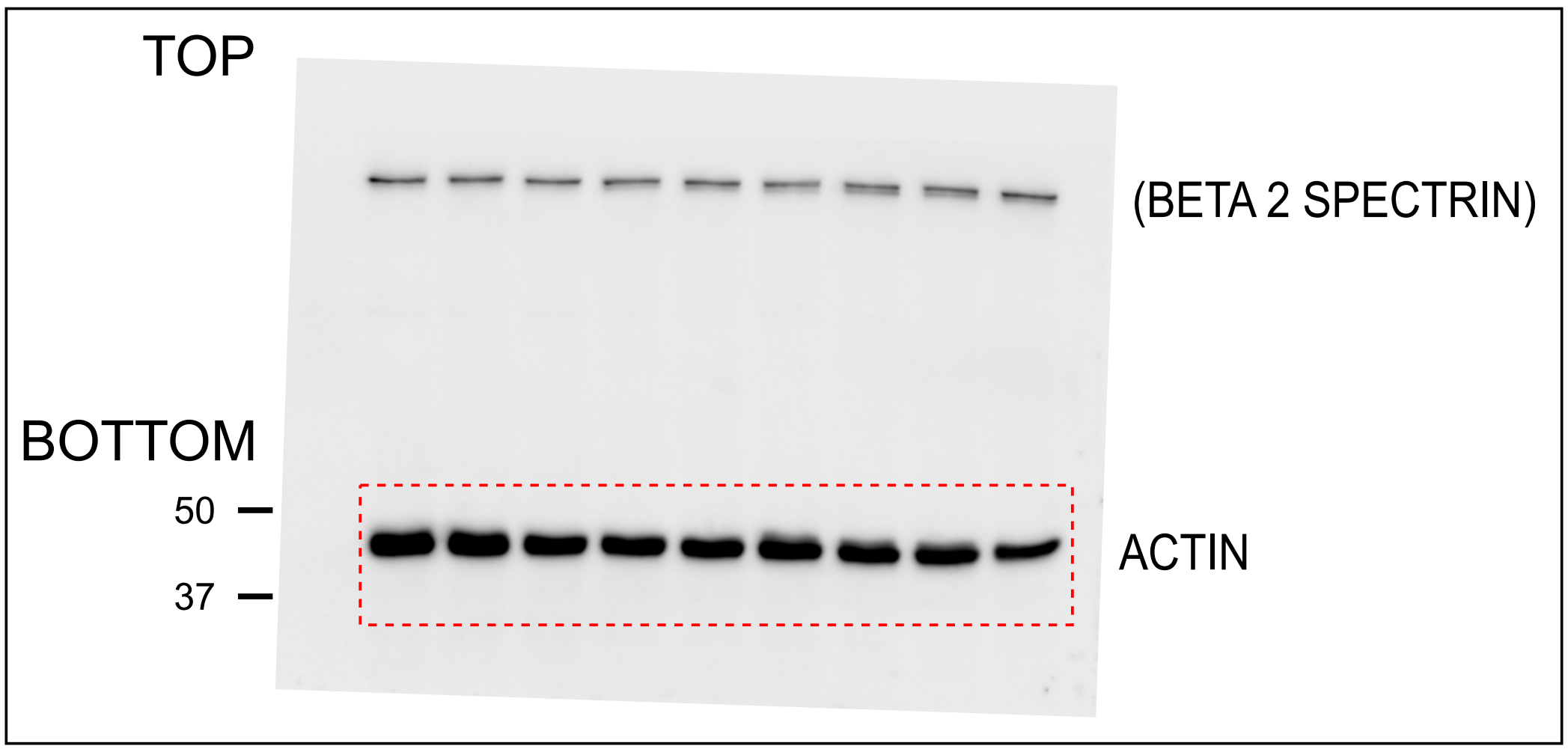

6 FIGURE 4 B

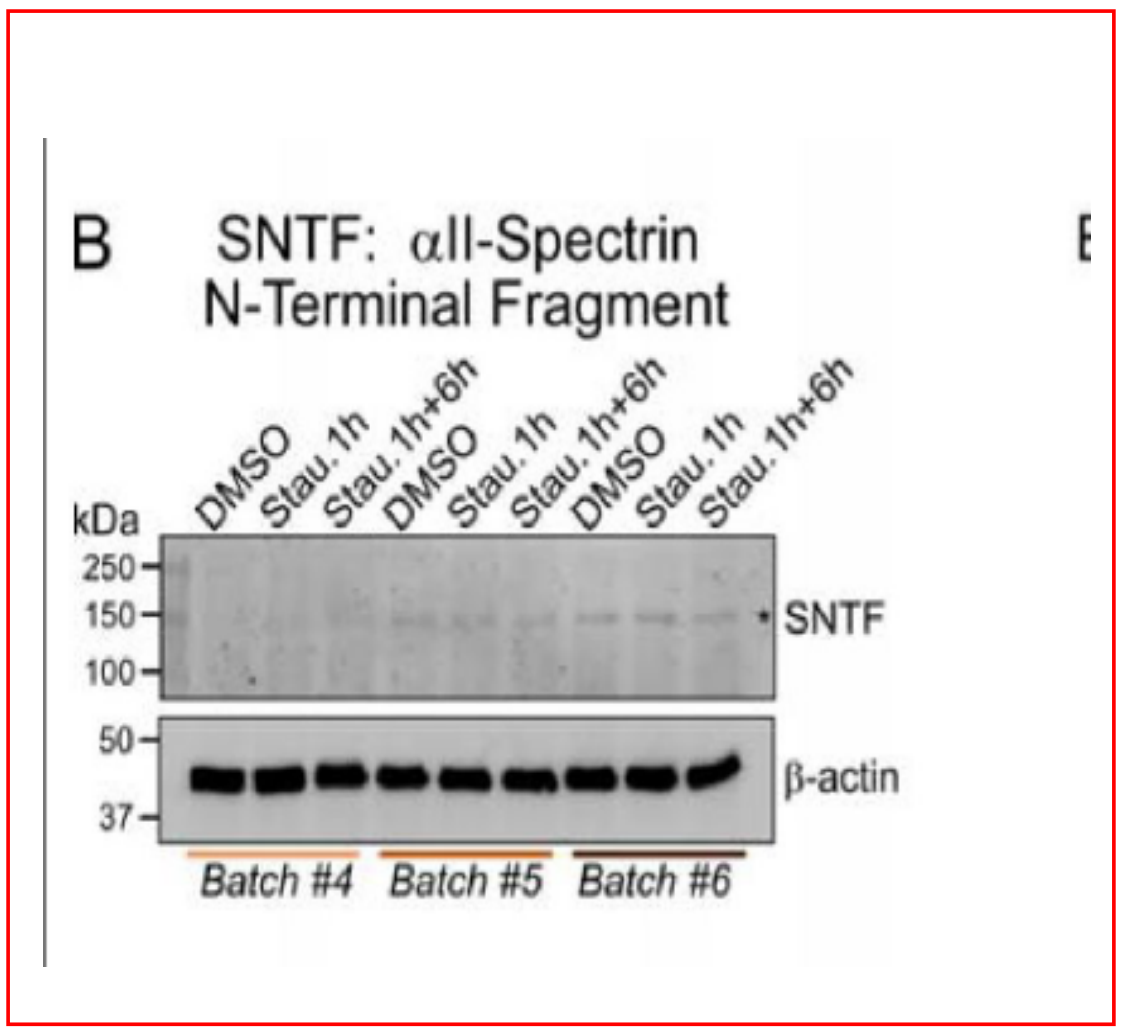

7

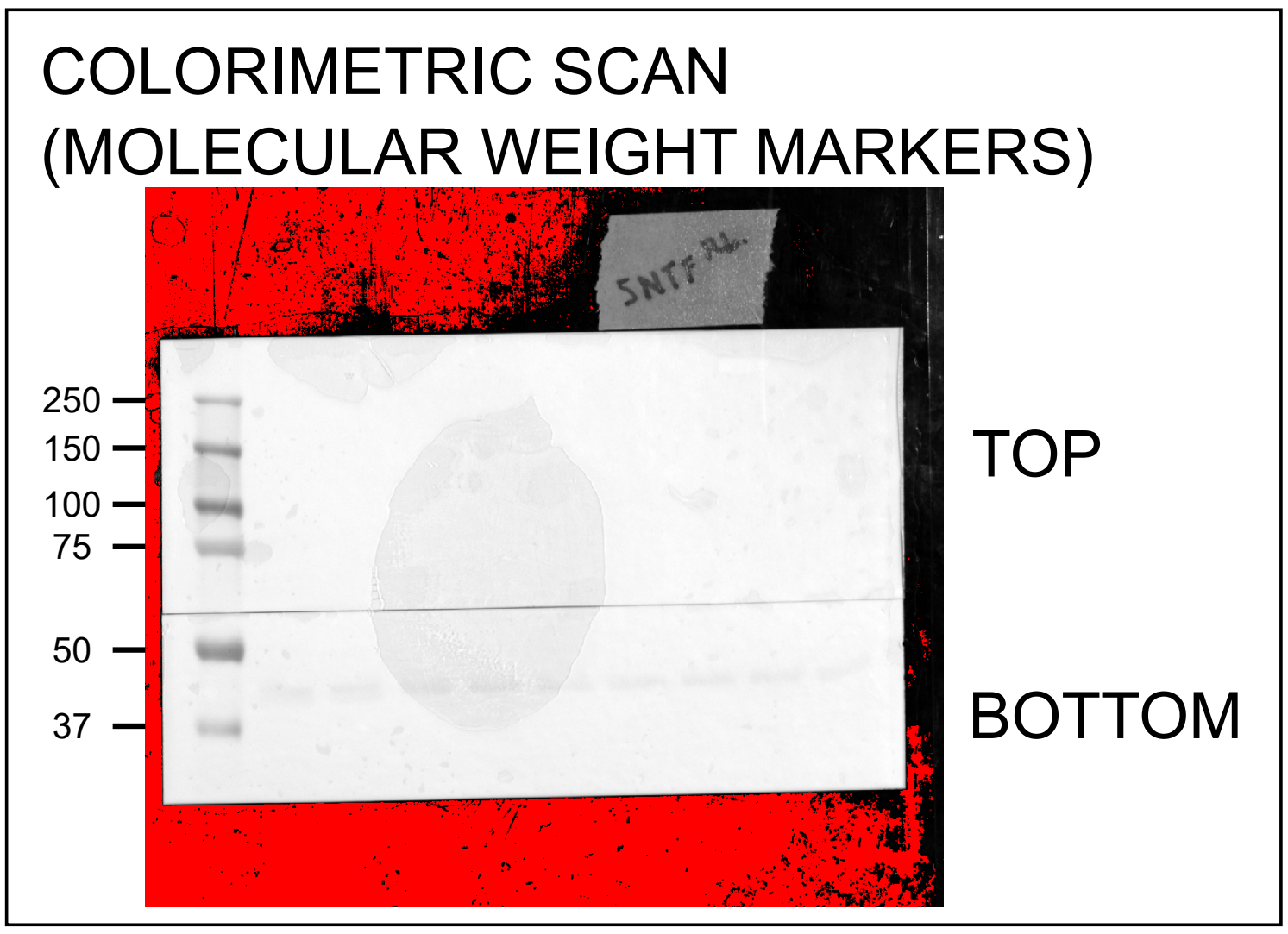

8

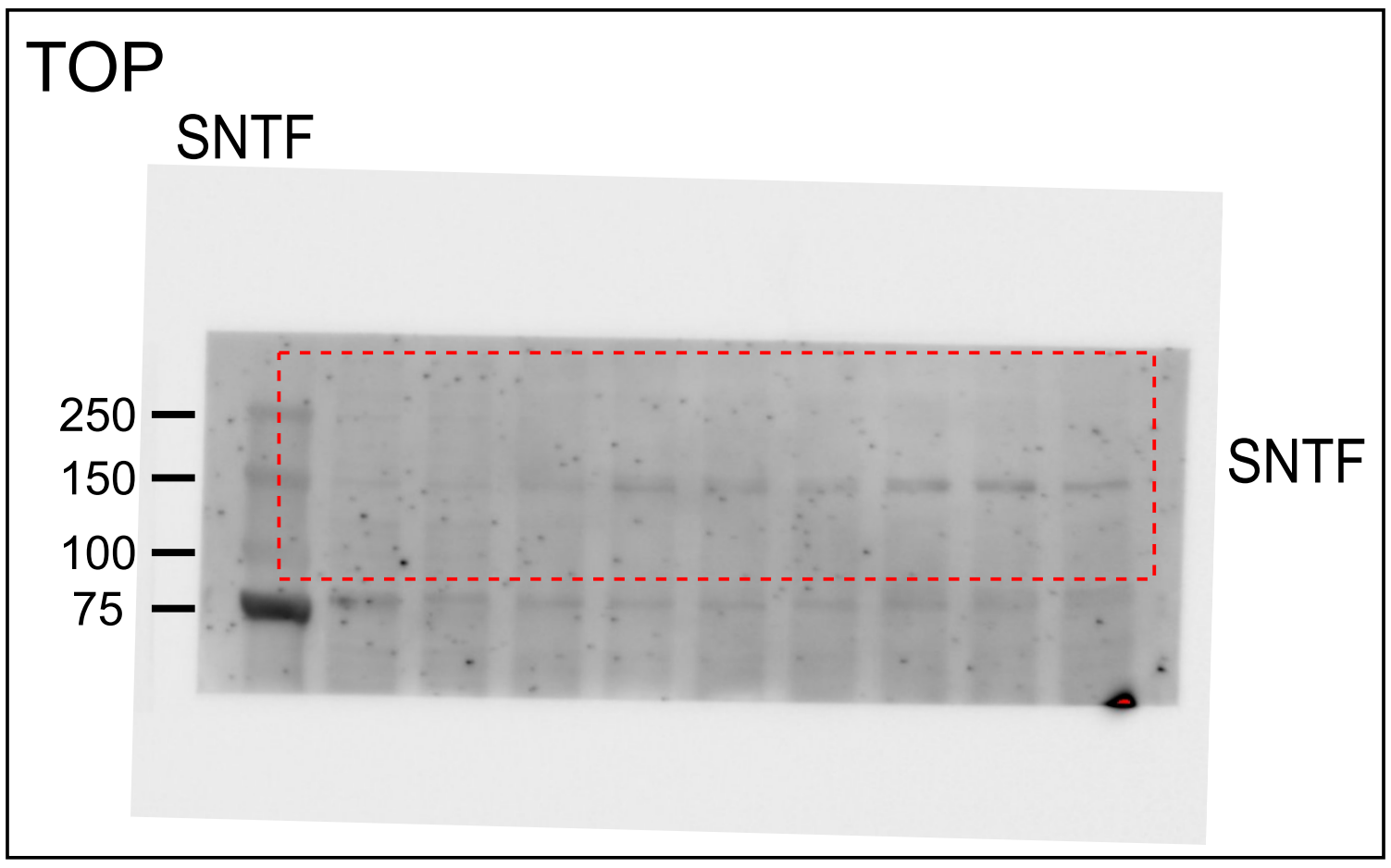

9

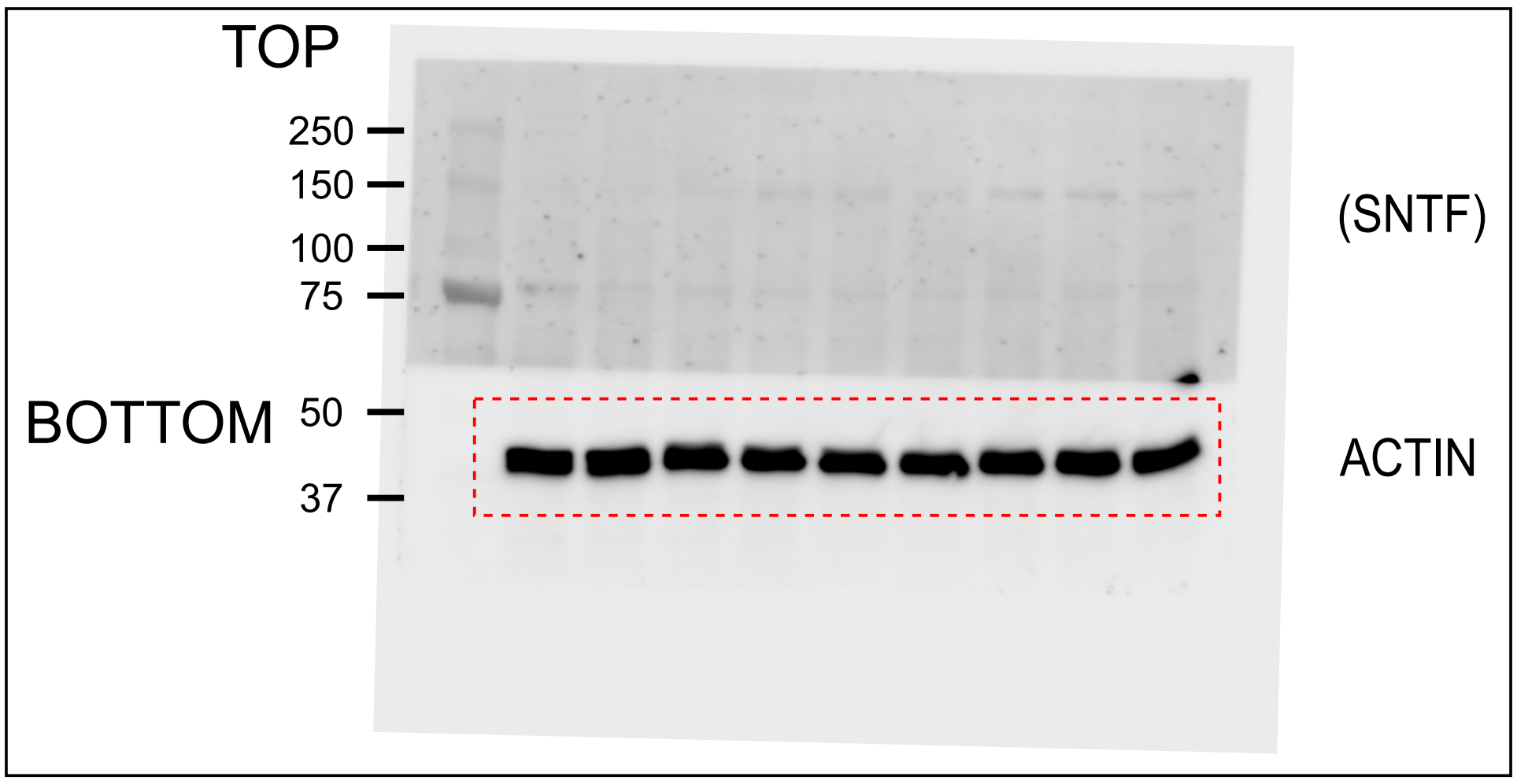

Supplement: Supplementary file 1 [file elife-108021-fig4-data1.pdf]
