# Supplementary material for: BetaII-spectrin gaps and patches emerge from the patterned assembly of the actin/spectrin membrane skeleton in human motor neuron axons
Source: eLife. 2026 Sep 8;14:RP108021. doi: 10.7554/eLife.108021 (PMC13553066; doi:10.7554/eLife.108021)

SUPPLEMENTARY FIGURE 4 - SOURCE DATA 1

1 Suppl FIGURE D

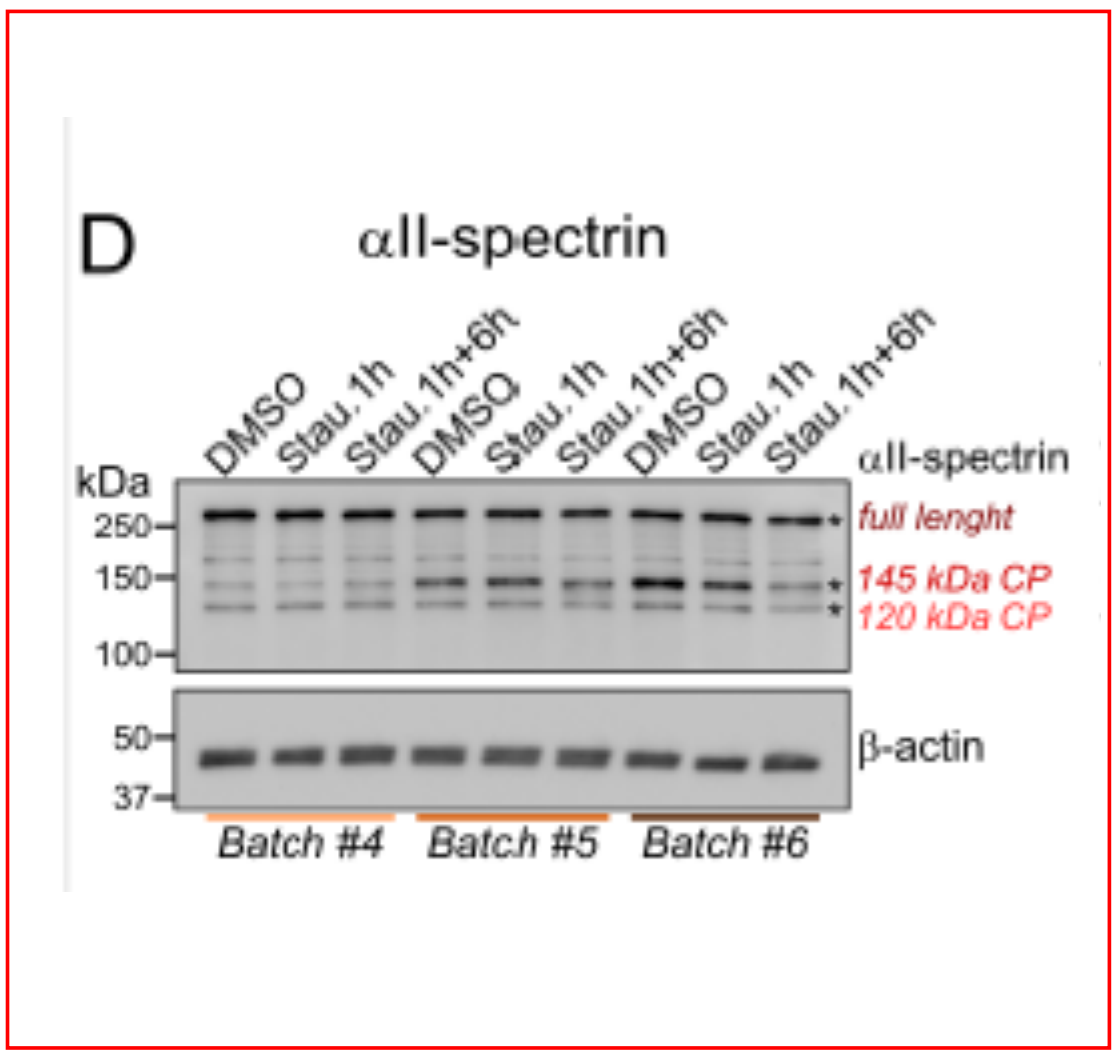

2

COLORIMETRIC SCAN
(MOLECULAR WEIGHT MARKERS)

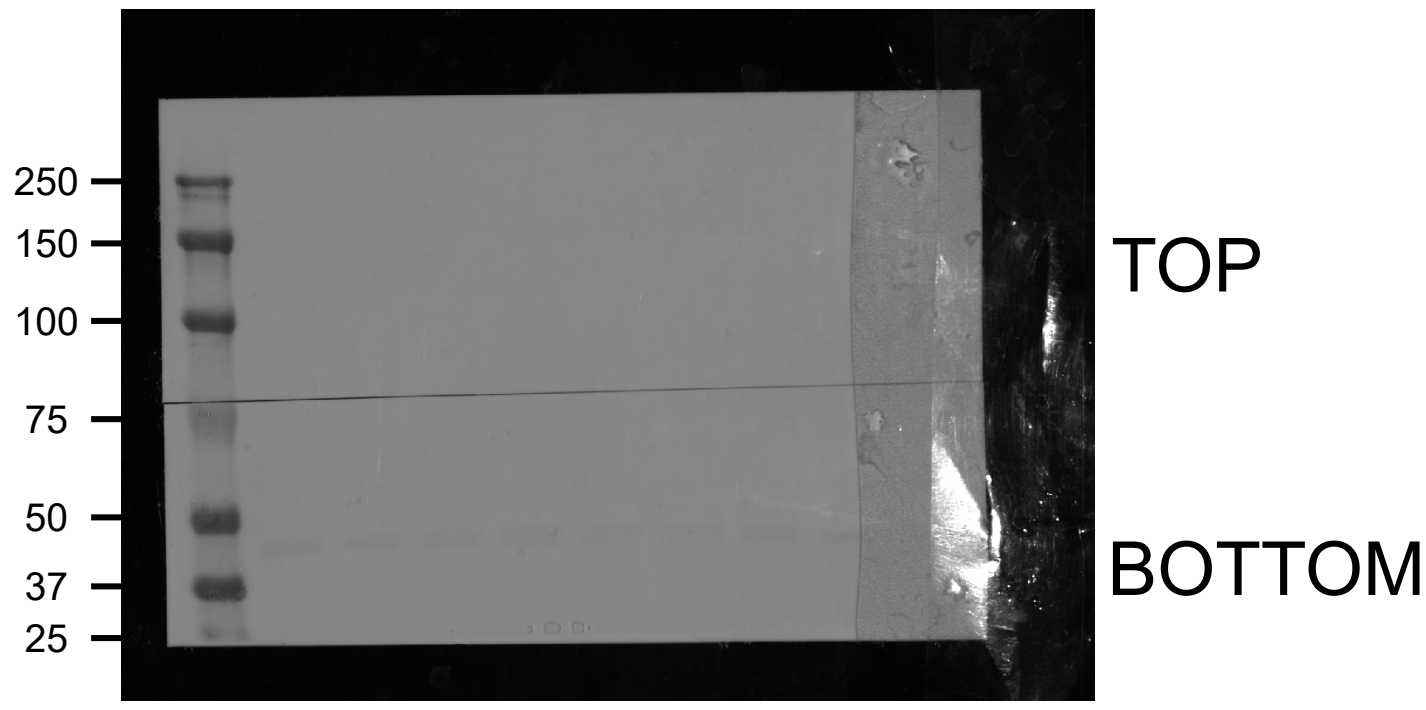

3

TOP
Alpha-2-spectrin

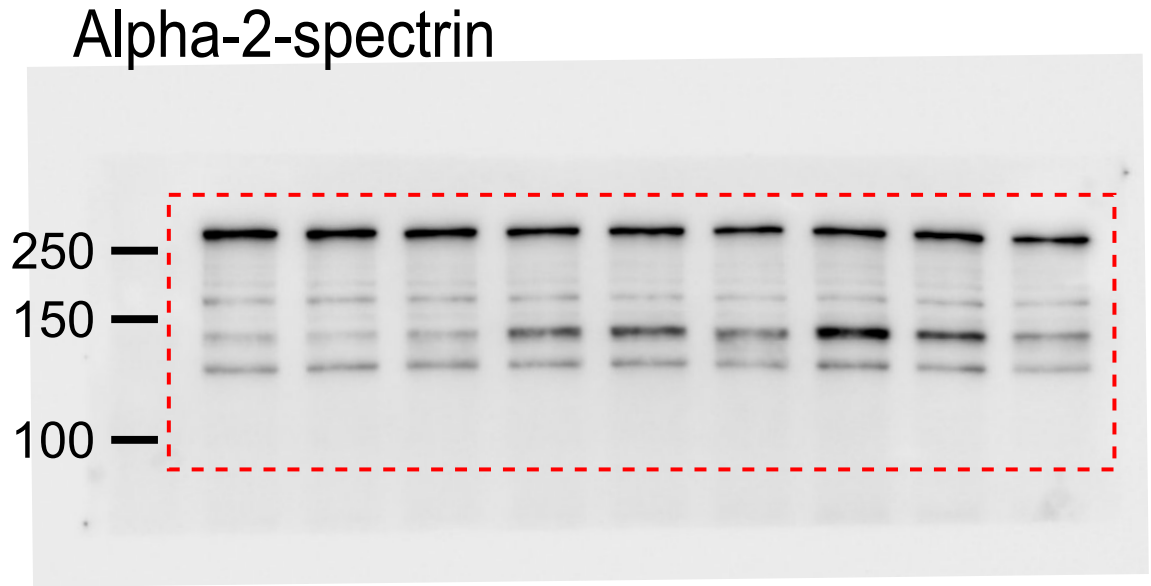

4

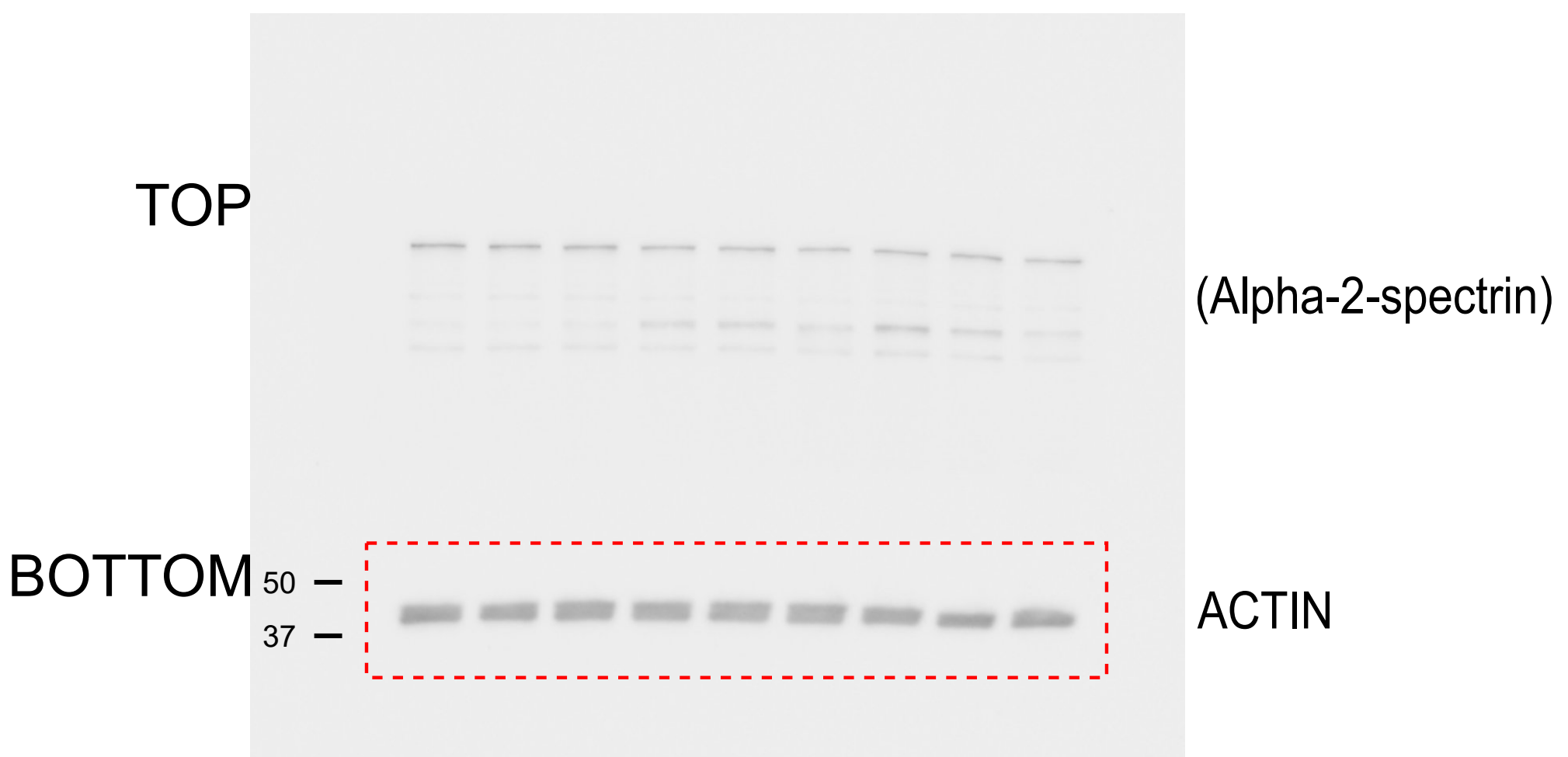

5 Suppl FIGURE 4 E

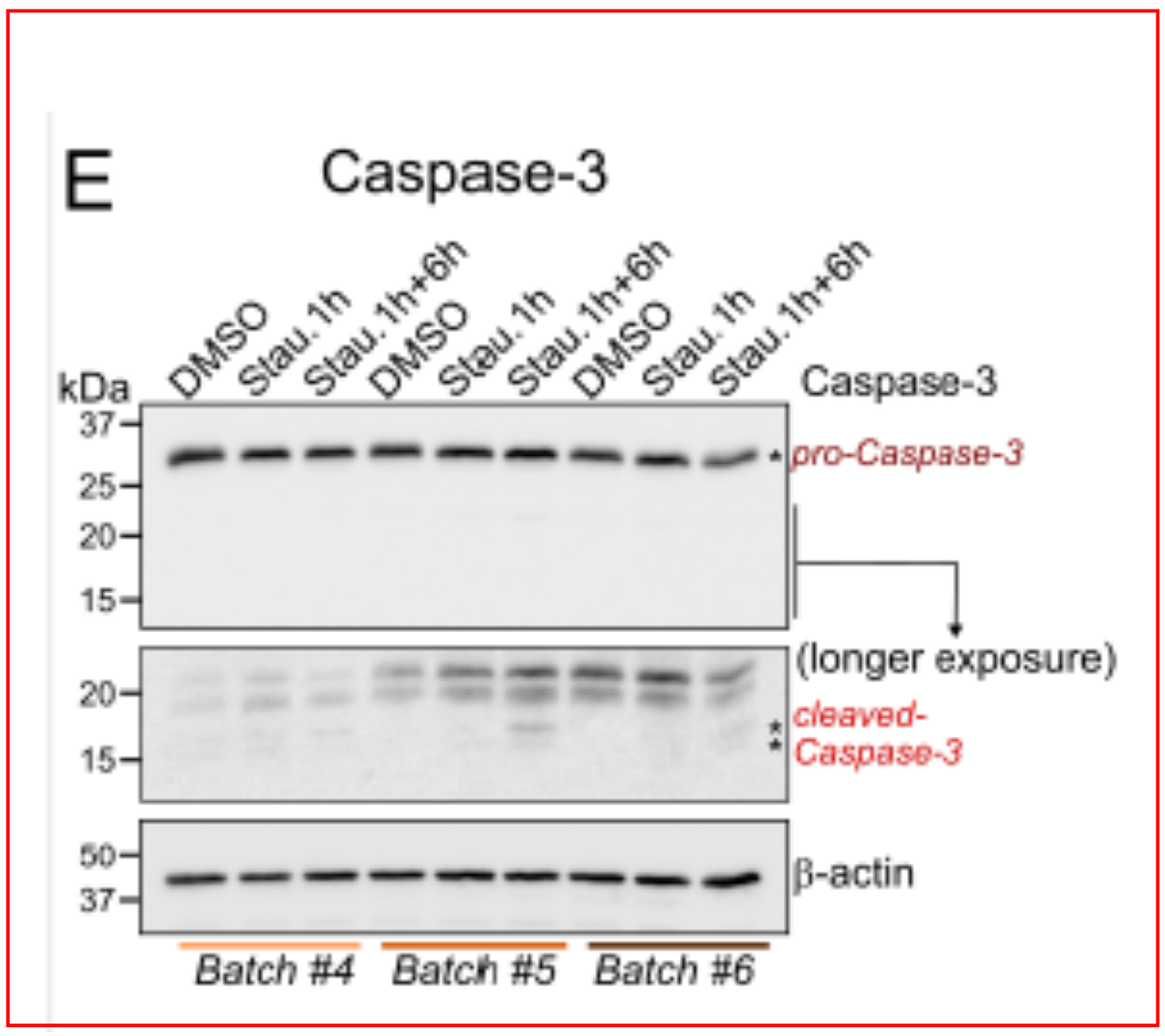

6

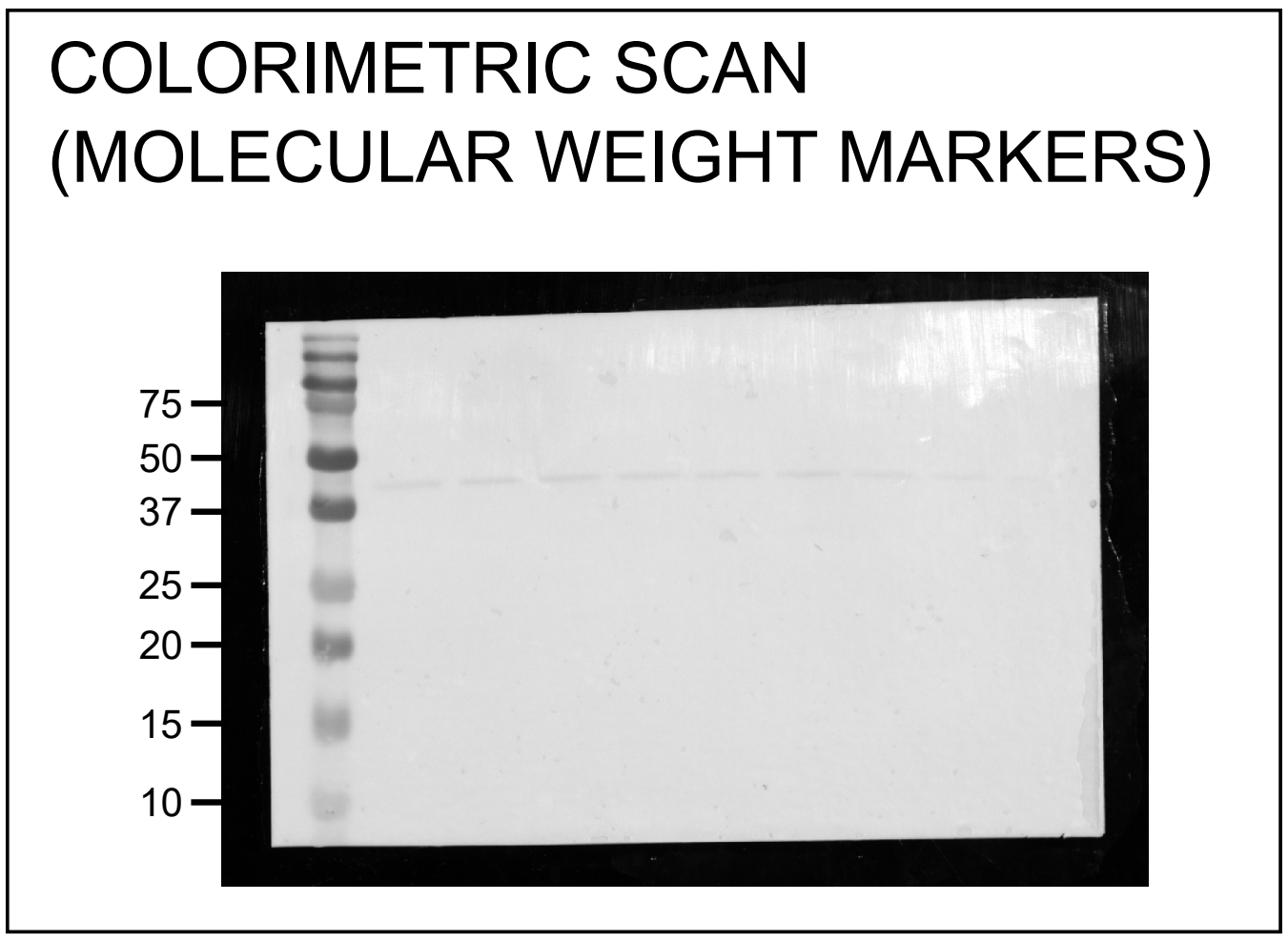

7

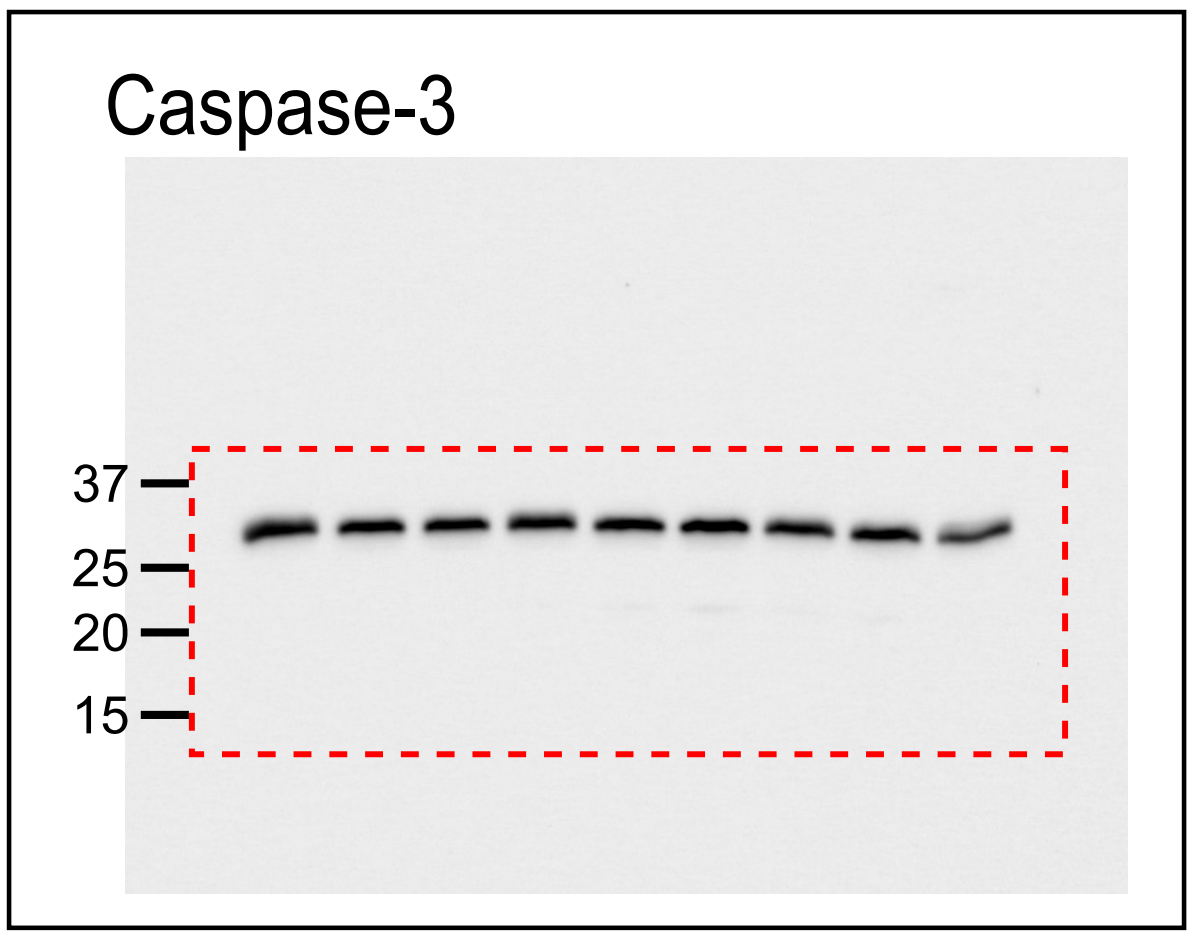

8

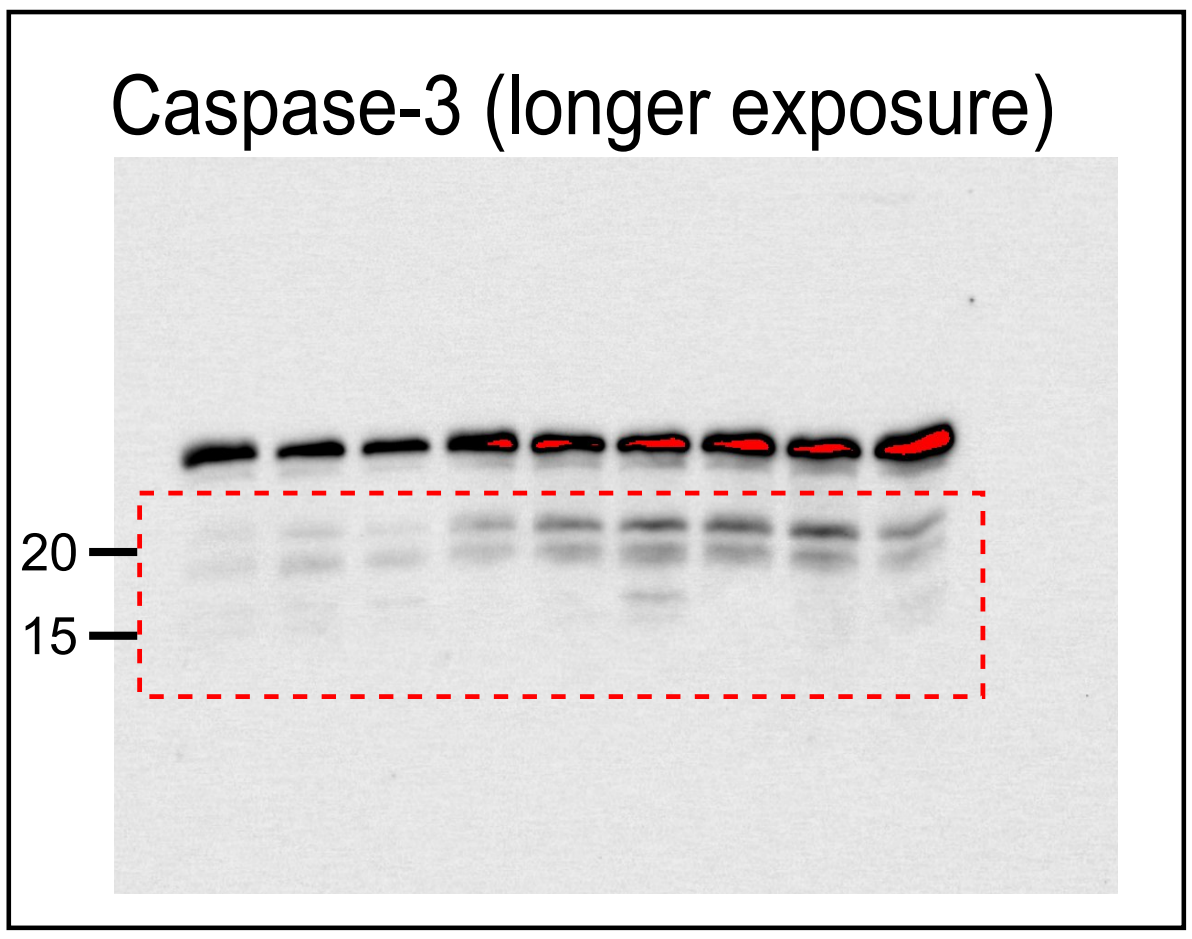

9

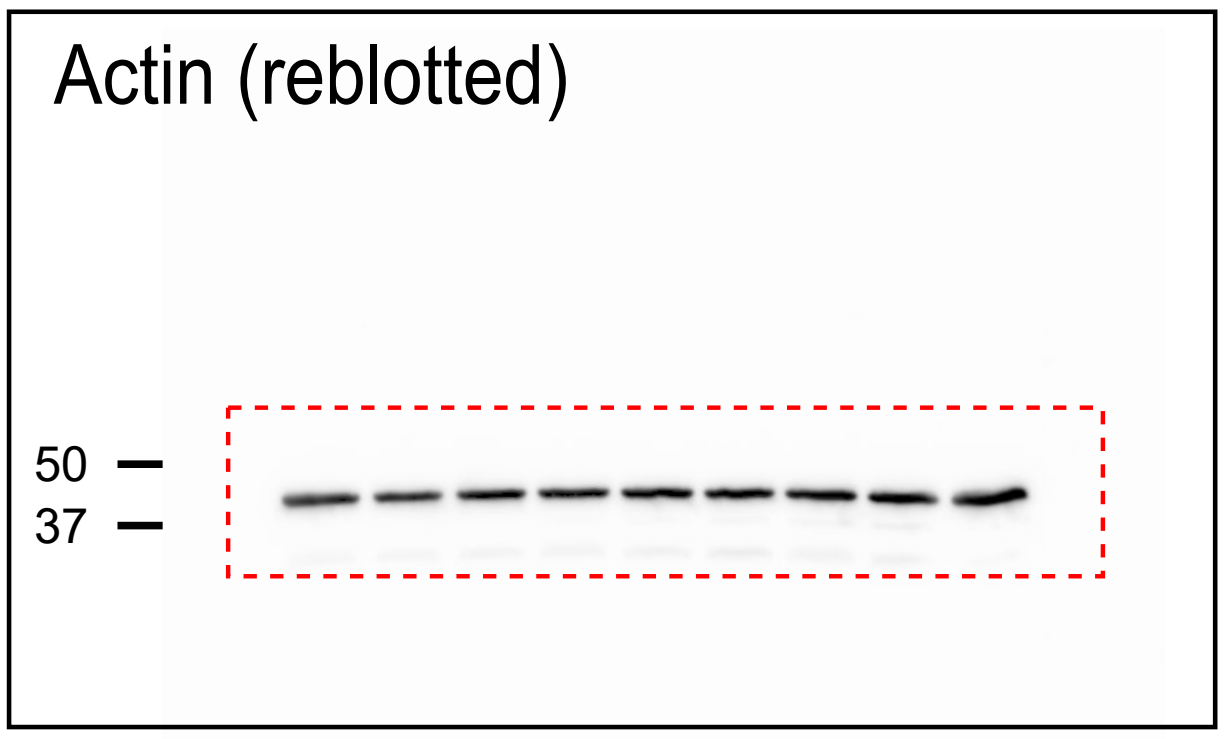

10 Suppl FIGURE 4 F

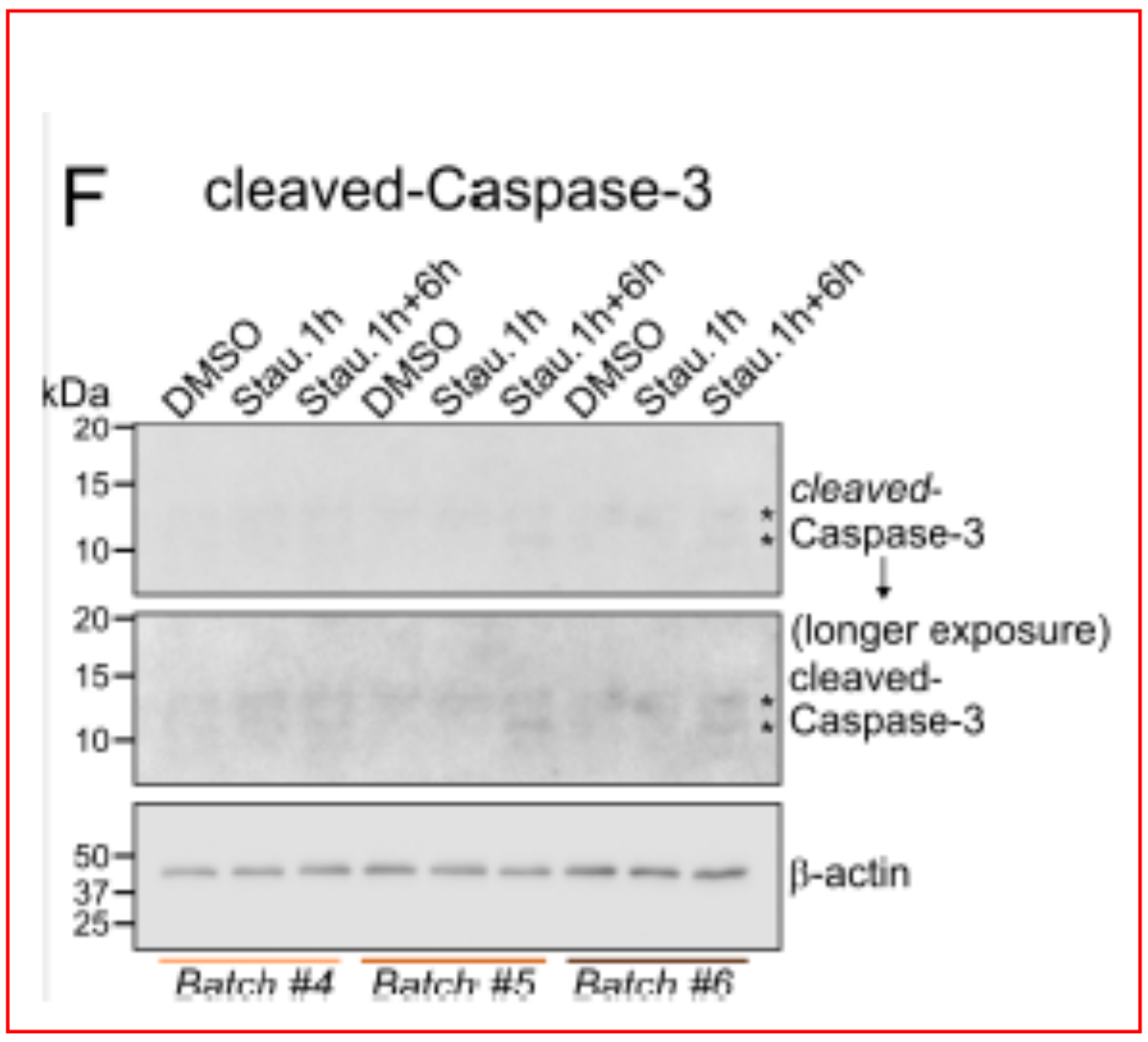

11

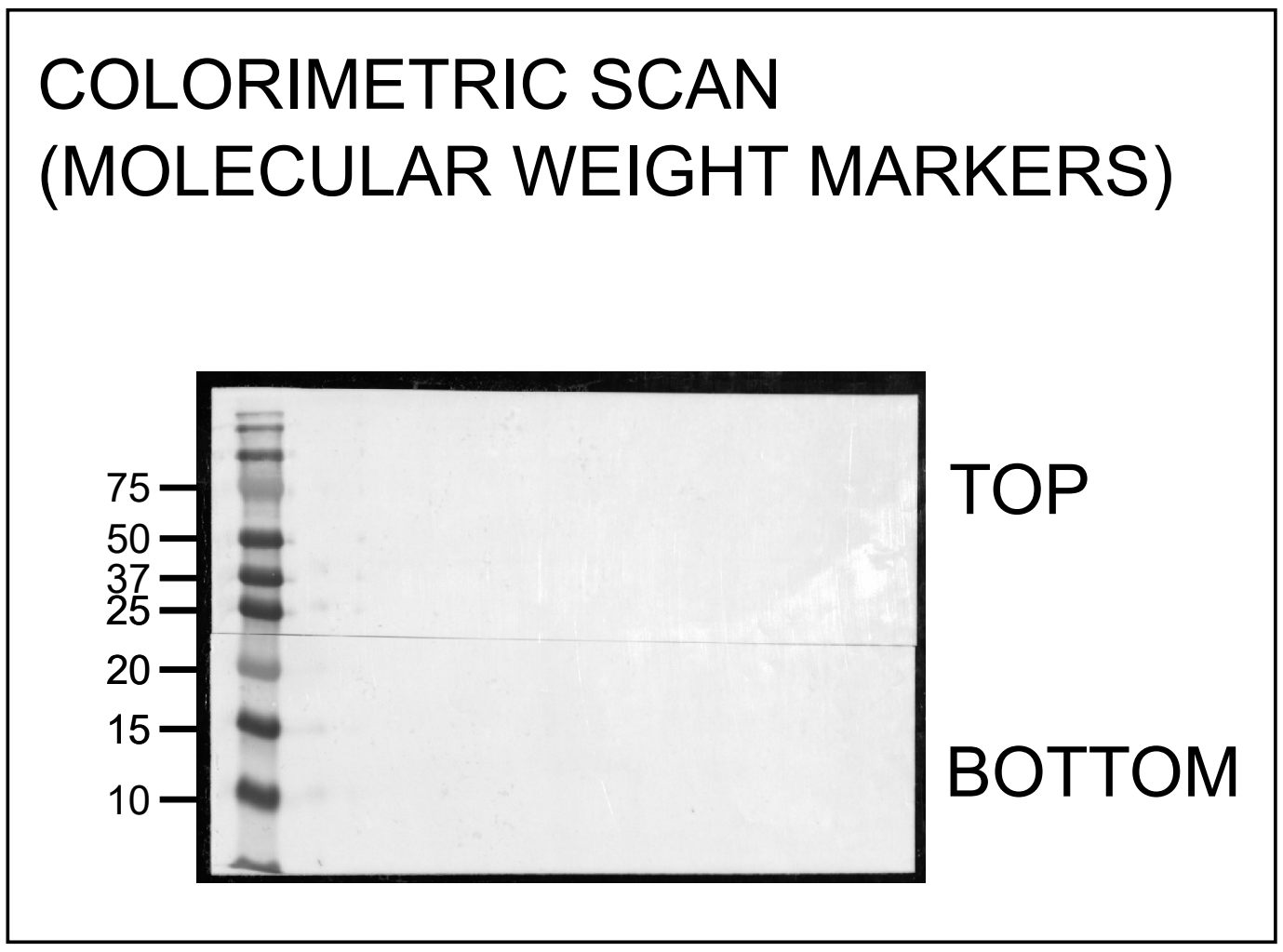

12

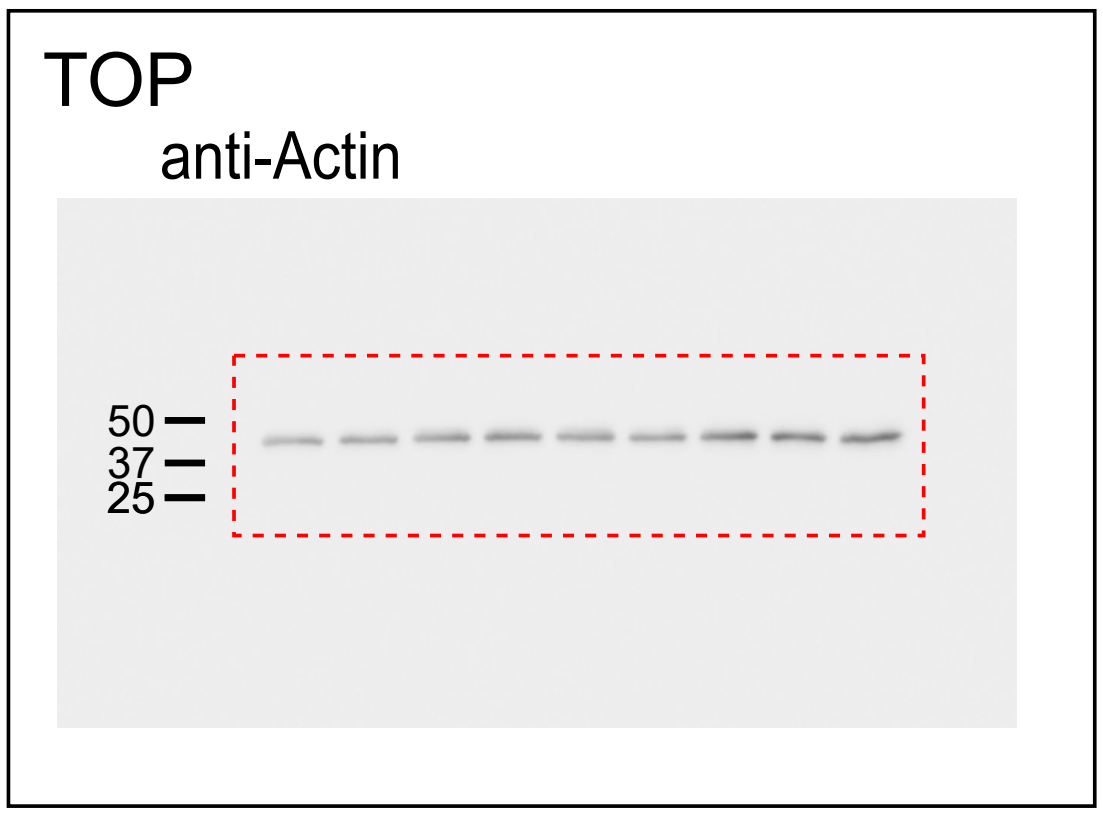

13

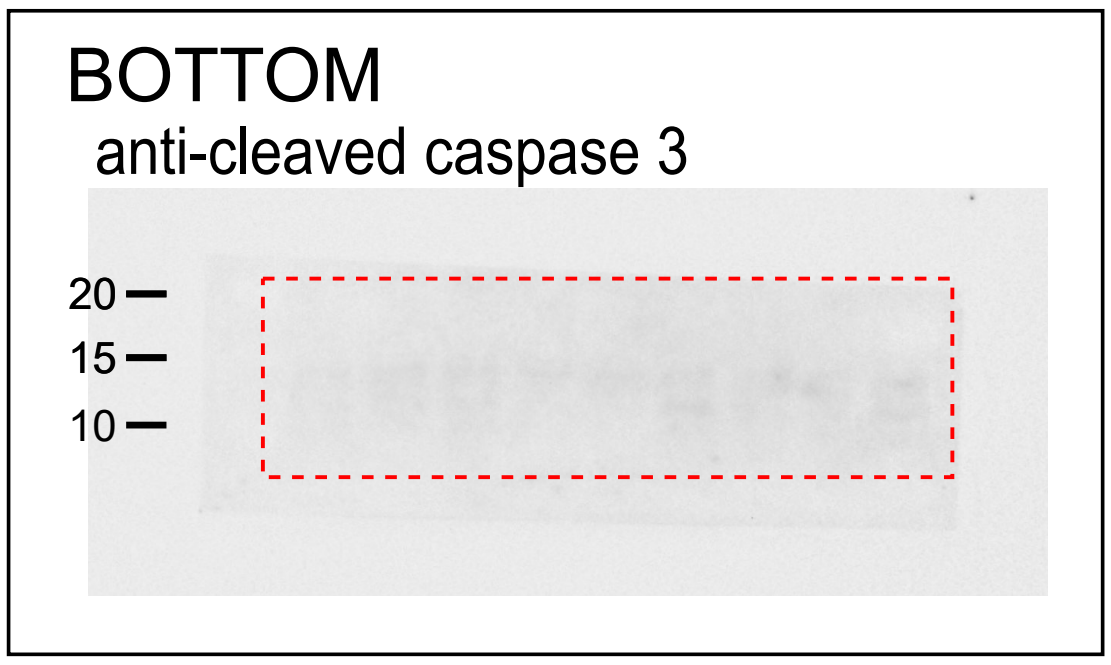

14

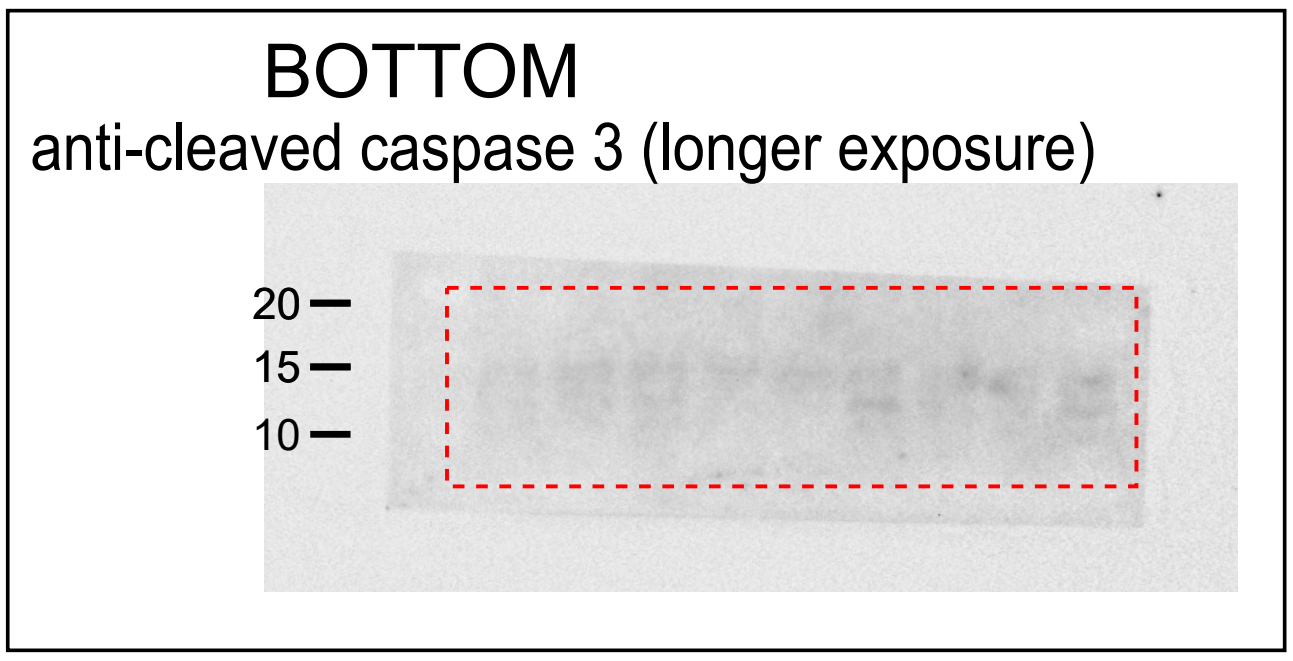

Supplement: Supplementary file 2 [file elife-108021-fig4-figsupp1-data1.pdf]
