# Supplementary material for: BetaII-spectrin gaps and patches emerge from the patterned assembly of the actin/spectrin membrane skeleton in human motor neuron axons
Source: eLife. 2026 Sep 8;14:RP108021. doi: 10.7554/eLife.108021 (PMC13553066; doi:10.7554/eLife.108021)
Supplement: Peer review history

# Editor report: eLife Assessment

**DOI:** 10.7554/eLife.108021.3.sa0

**Authors:** Pascal Martin

**Affiliations:** Institut Curie France

**Keywords:** Convincing, Valuable

This **valuable** study characterizes the emergence of the membrane-associated periodic cytoskeleton (MPS) in the axons of human motor neurons derived from induced pluripotent stem cells. Super-resolution imaging of beta-II spectrin provides **convincing** evidence for the patterned assembly of spectrin-poor gaps and spectrin-rich MPS in the medial region of the axons and its enhancement by the kinase inhibitor staurosporine. The data advocates against gap formation by axonal degeneration or cytoskeleton disassembly in a continuous MPS. Instead, a continuous MPS may result from nascent MPS patches and their maturation, a model that would benefit from live imaging for validation.

# Referee report: Reviewer #1 (Public review)

**DOI:** 10.7554/eLife.108021.3.sa1

The authors have presented a revised version of their investigation into the Membrane Associated Periodic Skeleton (MPS) in iPSC derived human motor neurons. As mentioned in the earlier report, the main observations reported in this article-occurrence of patch and gap arrangement of MPS-is very interesting. The real puzzle is whether, and if so how, this structure coarsens over time to produce continuous MPS.

Following suggestions from reviewers, the authors attempted live cell imaging, but the results were not consistent enough and the authors point out difficulties in obtaining sufficient numbers and possible artifacts of over-expression. This investigation would have been much stronger with live cell imaging data on the dynamics of patch and gap structures.

# Referee report: Reviewer #2 (Public review)

**DOI:** 10.7554/eLife.108021.3.sa2

Summary:

In this manuscript, Gazal et al., describe the presence of unique gaps and patches of BetaII-spectrin in medial sections of long human motor neuron axons. BII-spectrin, along with Alpha-spectrin forms horizontal linkers between 180nm spaced F-actin rings in axons. These F-actin rings along with the spectrin linkers form membrane periodic structures (MPS) which are critical for maintenance of the integrity, size and function of axons. The primary goal of the authors was to address if long motor axons, particularly those carrying familial mutations associated with the neurodegenerative disorder ALS, show defects in gaps and patches of BetaII-spectrin ultimately leading to degradation of these neurons.

Strengths:

The experiments are well designed and the authors have used the right methods and cutting-edge techniques to address the questions in this manuscript. The use of human motor neurons and the use of motor neurons with different familial ALS mutations is a strength. The use of isogenic controls is a positive. The induction of gaps and patches by the kinase inhibitor staurosporine and their rescue by Latrunculin A is novel and well executed. The use of biochemical assays to explore the role of calpains is appropriate and well designed. The use of STED imaging to define the periodicity of MPS in the gaps and patches of spectrin is a strength.

Weaknesses:

Primary weakness is the lack of rigorous evaluation to validate the proposed model of spectrin capture from the gaps into adjacent patches by the use of photobleaching and live-imaging. Another point is the lack of investigation into how gaps and patches change in axons carrying the familial ALS mutations as they age, since 2 weeks is not a timepoint when neurodegeneration is expected to start.

Comment on revised version.

The authors have given a point-by-point response to all the reviewer's concerns. They have also addressed concerns which I raised adequately. I have no further concerns.

# Referee report: Reviewer #3 (Public review)

**DOI:** 10.7554/eLife.108021.3.sa3

Summary:

Gazal et al present convincing evidence supporting a new model of MPS formation where a gap-and-patch MPS pattern coalesces laterally to give rise to a lattice covering the entire axon shaft.

Strengths:

(1) This is a very interesting study that supports a change in paradigm in the model of MPS lattice formation.

(2) Knowledge on MPS organization is mainly derived from studies using rat hippocampal neurons. In the current manuscript, Gazal et al use human IPS-derived motor neurons, a highly relevant neuron type to further the current knowledge on MPS biology.

(3) The quality of the images provided, specifically of those involving super-resolution is of high standards, supporting adequately the conclusions of the authors.

Weaknesses:

(1) The main concern raised by the manuscript is the assumption that staudosporine-induced gap and patch formation recapitulates the physiological assembly of gaps and patches of betaII-spectrin.

(2) One technical challenge that limits a more compelling support of the new model of MPS formation, is that fixed neurons are imaged, which precludes the observation of patch coalescence.

# Author comment: Author response

**DOI:** 10.7554/eLife.108021.3.sa4

**Authors:** Nahir Guadalupe Gazal, Maria Jose Castellanos-Montiel, Guillermina Bruno, Anna Kristina Franco-Flores, Sarah Lépine, Lale Gursu, Ghazal Haghi, Gilles Maussion, Wolfgang Reintsch, Fernando D Stefani, Agustín Anastasía, Mariano Bisbal, Ezequiel Axel Gorostiza, Thomas M Durcan, Nicolás Unsain

**Affiliations:** https://ror.org/056tb7j80 Universidad Nacional de Córdoba Cordoba Argentina; McGill University Montreal Canada; https://ror.org/056tb7j80 Universidad Nacional de Córdoba Cordoba Argentina; McGill University Montreal Canada; McGill University Montreal Canada; McGill University Montreal Canada; Early Drug Discovery Unit (EDDU), The Neuro-Montreal Neurological Institute and Hospital, Department of Neurology and Neurosurgery, McGill University Edinburgh Canada; Early Drug Discovery Unit (EDDU), The Neuro-Montreal Neurological Institute and Hospital, Department of Neurology and Neurosurgery, McGill University MONTREAL Canada; McGill University Montreal Canada; Centro de Investigaciones en Bionanociencias (CIBION), Consejo Nacional de Investigaciones Cientificas y Tecnicas (CONICET), Godoy Cruz 2390, Ciudad Autonoma de Buenos Aires, C1425FQD, Argentina. Buenos Aires Argentina; https://ror.org/056tb7j80 Universidad Nacional de Córdoba Cordoba Argentina; Instituto de Investigación Médica Mercedes y Martín Ferreyra (INIMEC), Consejo Nacional de Investigaciones Científicas y Técnicas (CONICET), Universidad Nacional d Córdoba Argentina; Institute of Zoology, Biocenter Cologne, University of Cologne Cologne Germany; McGill University Montreal Canada; https://ror.org/056tb7j80 Universidad Nacional de Córdoba Cordoba Argentina

The following is the authors’ response to the original reviews.

**eLife Statement**

This valuable study characterizes the emergence of the membrane-associated periodic cytoskeleton (MPS) in the axons of human motor neurons derived from induced pluripotent stem cells. Super-resolution imaging of beta-II spectrin provides convincing evidence for the patterned assembly of spectrin-poor gaps and spectrin-rich MPS in the medial region of the axons and its enhancement by the kinase inhibitor staurosporine. The data advocates against gap formation by cytoskeleton disassembly in a continuous MPS. Instead, a continuous MPS may result from nascent MPS patches and their maturation, a model that would benefit from live imaging for validation.

(R1) We thank the reviewers and editor for their constructive and thoughtful feedback. We are pleased the reviewers found our evidence to be convincing and that our study provides a valuable framework for understanding the complex dynamics of MPS assembly.

**Public Reviews:**

**Reviewer #1 (Public review):**

Summary:

Ever since the surprising discovery of the membrane-associated Periodic Skeleton (MPS) in axons, a significant body of published work has been aimed at trying to understand its assembly mechanism and function. Despite this, we still lack a mechanistic understanding of how this amazing structure is assembled in neuronal cells. In this article, the authors report a "gap-and-patch" pattern of labelled spectrin in iPSC-derived human motor neurons grown in culture. The mid-sections of these axons exhibit patches with reasonably well-organized MPS that are separated by gaps lacking any detectable MPS and having low spectrin content. Further, they report that the intensity modulation of spectrin is correlated with intensity modulations of tubulin as well. However, neurofilament fluorescence does not show any correlation. Using DIC imaging, the authors show that often the axonal diameter remains uniform across segments, showing a patch-gap pattern. Gaps are seen more abundantly in the midsection of the axon, with the proximal section showing continuous MPS and the distal segment showing continuous spectrin fluorescence but no organized MPS. The authors show that spectrin degradation by caspase/calpain is not responsible for gap formation, and the patches are nascent MPS domains. The gap and patch pattern increases with days in culture and can be enhanced by treating the cells using the general kinase inhibitor staurosporine. Treatment with the actin depolymerizing agent Latrunculin A reduces gap formation. The reasons for the last two observations are not well understood/explained.

(R2) We thank the reviewer for the detailed and accurate description of the data shown and its relevance to further our understanding of MPS assembly mechanism and function.

Strengths:

The claims made in the paper are supported by extensive imaging work and quantification of MPS. Overall, the paper is well written and the findings are interesting. Although much of the reported data are from axons treated with staurosporine, this may be a convenient system to investigate the dynamics of MPS assembly, which is still an open question.

(R3) We thank the reviewer for the positive comments on the manuscript and the convenience of the experimental system developed to further study the dynamics of MPS assembly. We hope others turn into motor neurons to explore cortical cytoskeleton biology and hopefully shed light into their susceptibility in various degenerative diseases.

Weaknesses:

Much of the analysis is on staurosporine-treated cells, and the effects of this treatment can be broad. The increase in patch-gap pattern with days in culture is intriguing, and the reason for this needs to be checked carefully. It would have been nice to have live cell data on the evolution of the patch and gap pattern using a GFP tag on spectrin. The evolution of individual patches and possible coalescence of patches can be observed even with confocal microscopy if live cell super-resolution observation is difficult.

(R4) Because staurosporine may hit various kinases relevant to the phenomenon under study we did not elaborate too deeply on the likely targets in the discussion. We have, however, included the possibility that the relevant kinase in this matter could be PKC, in light of the new study published while our manuscript was under revision (Heller et al., 2025) (see second last paragraph in the Discussion section). Staurosporine represented a convenient initial approach that allowed us to find the phenomenon, and we are now conducting new studies dissecting the molecular pathways involved. However, the extent of such studies lies beyond the scope of the present report.

See R16 regarding possible live-imaging experiments using tagged βII-spectrin constructs.

Some more comments:

(1) Axons can undergo transient beading or regularly spaced varicosity formation during media change if changes in osmolarity or chemical composition occur. Such shape modulations can induce cytoskeletal modulations as well (the authors report modulations in microtubule fluorescence). The authors mention axonal enlargements in some instances. Although they present DIC images to argue that the axons showing gaps are often tubular, possible beading artefacts need to be checked. Beading can be transient and can be checked by doing media changes while observing the axons on a microscope.

(R5) As we acknowledge this possibility, we believe that, even if they occurred, they could not contribute to our observations of gaps-and-patches phenomenon since this latter subsisted long (hours and days) after any gross manipulation of media. Moreover fixed samples, when observed under DIC, confocal or STED did not evidence such beadings. We do refer to a characteristic local enlargement that was very localized and very low in numbers (see Fig.1C and E, and Suppl. Fig1C and E), so we don't believe these are transient, and do not resemble the structure referred to as beading. Structurally, beading is essentially different since it appears in rows of consecutive “beads” in long stretches, where round, small enlargements of axonal caliber are arranged in a consecutive manner, resembling pearls on a string. As mentioned by the reviewer, the beading phenomena can occur transiently when drastically changing media osmolarity (rarely done in cell culture manipulations) or non-tranciently when axons are undergoing degeneration. Indeed, to prevent gross changes in osmolarity, our routine fixation is a 4% PFA and 4% sucrose in PBS. In any case, we did not observe signs of beading in the cultures used for this study.

(2) Why do microtubules appear patchy? One would imagine the microtubule lengths to be greater than the patch size and hence to be more uniform.

(R6) Our stainings are for tubulin protein isoforms beta-III and alpha-II. That is, they would label microtubules, but free tubulin as well. Hence we don't think this is evidence for “patchy microtubules”. The slight decrease in intensity for tubulin within gaps is indeed something to investigate, and can indicate that tubulin prefers to accumulate within patches.

(3) Why do axons with gaps increase with days in culture? If patches are nascent MPS that progressively grow, one would have expected fewer gaps with increasing days in culture. Is this indicative of some sort of degeneration of axons?

(R7) We agree with the apparent discrepancy. However, one has to take into account that these axons are still elongating even at 2 weeks in culture and beyond. Hence, at any time point, there is a new axonal compartment recently added, and hence, with low βII-spectrin and no organized MPS. Also, the dynamical evolution of the gaps-and-patches structure has to take into account the rate of βII-spectrin supply and transport. If supply is somehow lower than a given threshold, it is expected that there will be more gaps, given the new, more distant parts of the axons have a lower supply of βII-spectrin. To explore this formally, we are working on simulations of these multifactorial dynamic systems to better understand this, that together with key experimental observations would enhance our understanding into our model of MPS assembly in growing axons. However, findings for this project will be the subject of another manuscript.

(4) It is surprising that Latrunculin A reduces gap formation induced by staurosporine (also seems to increase MPS correlation) while it decreases actin filament content. How can this be understood? If the idea is to block actin dynamics, have the authors tried using Jasplakinolide to stabilize the filaments?

(R8) The results with the co-treatment with Latrunculin A and Staurosporine are indeed intriguing, and provide clear evidence that the gap-and-patch pattern arises from local assembly of the MPS, requiring newly formed actin filaments. On the other hand, the fact that F-actin within the pre-formed MPS seems unaffected is not surprising. There are many different populations of F-actin in axons (i.e. MPS rings, longitudinal filaments, actin patches, actin trails), all of which have a different rate of monomer turnover. Latrunculin A affects filaments indirectly. The target of Latrunculin A is not actin filaments, but free monomers. Monomer sequestration ultimately affects actin filaments: filaments are constantly exchanging monomers, but, devoid of free monomers, filaments get shorter and eventually disappear. The drastic decrease in global F-actin in LatA-treated axons reflects that. The fact that F-actin in the MPS is preserved shows that these filaments are stable -if they are not losing monomers in the time frame of the treatment, the filament remains unaffected. This subject is extensively covered in the 8th paragraph of the Discussion section.

We have not used Jasplakinolide. The expected outcome will not mimic that of Latrunculin A since Jasplakinolide has a different mechanism of action (i.e. it binds -and stabilizes- the actin filament).

(5) The authors speculate that the patches are formed by the condensation of free spectrins, which then leaves the immediate neighborhood depleted of these proteins. This is an interesting hypothesis, and exploring this in live cells using spectrin-GFP constructs will greatly strengthen the article. Will the patch-gap regions evolve into continuous MPS? If so, do these patches expand with time as new spectrin and actin are recruited and merge with neighboring patches, or can the entire patch "diffuse" and coalesce with neighboring patches, thus expanding the MPS region?

(R9) We agree with the reviewer's interpretation. A virtue of our experimental model and our interpretations of the observations in fixed cells is that it gives rise to informative questions such as the ones posed by the reviewer. See R16 regarding possible live-imaging experiments using tagged βII-spectrin constructs.

**Reviewer #2 (Public review):**

Summary:

In this manuscript, Gazal et al. describe the presence of unique gaps and patches of BetaII-spectrin in medial sections of long human motor neuron axons. BII-spectrin, along with Alpha-spectrin, forms horizontal linkers between 180nm spaced F-actin rings in axons. These F-actin rings, along with the spectrin linkers, form membrane periodic structures (MPS) which are critical for the maintenance of the integrity, size, and function of axons. The primary goal of the authors was to address whether long motor axons, particularly those carrying familial mutations associated with the neurodegenerative disorder ALS, show defects in gaps and patches of BetaII-spectrin, ultimately leading to degradation of these neurons.

(R10) We thank the reviewer for the detailed and accurate description of the data shown.

Strengths:

The experiments are well-designed, and the authors have used the right methods and cutting-edge techniques to address the questions in this manuscript. The use of human motor neurons and the use of motor neurons with different familial ALS mutations is a strength. The use of isogenic controls is a positive. The induction of gaps and patches by the kinase inhibitor staurosporine and their rescue by Latrunculin A is novel and well-executed. The use of biochemical assays to explore the role of calpains is appropriate and well-designed. The use of STED imaging to define the periodicity of MPS in the gaps and patches of spectrin is a strength.

(R11) We thank the reviewer for the positive comments on the manuscript, the techniques used and the proposed model.

Weaknesses:

The primary weakness is the lack of rigorous evaluation to validate the proposed model of spectrin capture from the gaps into adjacent patches by the use of photobleaching and live imaging. Another point is the lack of investigation into how gaps and patches change in axons carrying the familial ALS mutations as they age, since 2 weeks is not a time point when neurodegeneration is expected to start.

(R12) See R16 regarding possible live-imaging experiments using tagged βII-spectrin constructs.

We don't discard the notion that axons carrying familial ALS mutations will show defects in MPS formation and/or stability when observed at longer culture times, or under culture conditions that promote neuronal aging (Guix et al., 2021). Thus, we continue to work with these cells, but the goal of such project lies well beyond the primary message of the present manuscript, as we discuss in the second paragraph of the Discussion section.

**Reviewer #3 (Public review):**

Summary:

Gazal et al present convincing evidence supporting a new model of MPS formation where a gap-and-patch MPS pattern coalesces laterally to give rise to a lattice covering the entire axon shaft.

Strengths:

(1) This is a very interesting study that supports a change in paradigm in the model of MPS lattice formation.

(2) Knowledge on MPS organization is mainly derived from studies using rat hippocampal neurons. In the current manuscript, Gazal et al use human IPS-derived motor neurons, a highly relevant neuron type, to further the current knowledge on MPS biology.

(3) The quality of the images provided, specifically of those involving super-resolution, is of a high standard. This adequately supports the conclusions of the authors.

(R13) We thank the reviewer for the positive comments on the manuscript, the techniques used and the proposed model.

Weaknesses:

(1) The main concern raised by the manuscript is the assumption that staudosporine-induced gap and patch formation recapitulates the physiological assembly of gaps and patches of betaII-spectrin.

(R14) Along the project, various gaps-and-patches parameters were measured in different conditions and stainings. In all these examinations the only parameter that changed considerably was their abundance. While this suggests that the gaps-and-patches features are comparable between control and staurosporine-treated cells, we acknowledge as a general caution regarding negative data—that subtle qualitative differences cannot be entirely ruled out. We have now emphasized this possibility in the 9th paragraph of the Discussion section.

(2) One technical challenge that limits a more compelling support of the new model of MPS formation is that fixed neurons are imaged, which precludes the observation of patch coalescence.

(R15) See R16 regarding possible live-imaging experiments using tagged βII-spectrin constructs.

**Recommendations for the authors:**

**Reviewing Editor Comments:**

The reviewers all agree that the work would strongly benefit from live imaging to assess the maturation dynamics of the gap/patch pattern.

(R16) Reviewers agreed that some of the conclusions of our manuscript would benefit from live imaging for validation. Various anticipated technical and biological challenges made these approaches not to be conducted for this initial study on human motor neurons. Just to mention the most important, from previous work of our labs, these cells themselves are difficult to transfect at 2 weeks in culture. Also, ectopically expression of tagged βII-spectrin escapes normal expression control and it has been noticed that ectopic expression yields to protein localization that does not necessarily reflect the endogenous distribution, or that produces cellular responses that precludes the observation of the phenomena under study. These difficulties in studying over-expressed tagged βII-spectrin have been reported in the field, with mentions that the analysed axons were those expressing “low levels of the construct” (Boyer et al., 2026; Zhong et al., 2014; Zhou et al., 2022). Taking this into account, we did not anticipate that, for the goals of the present project, live-imaging was to be included. However, given the positive comments and reception of our conclusions, we sought to try to perform this challenging and risky approach. To that end, we used a C-terminus tagged mouse βII-spectrin-GreenLantern plasmid to transfect our cells (a kind gift from Dr. Subjohit Roy, UCSD, USA). After 3 rounds of differentiating cells and trying various combinations of plasmid quantity, lipofectimine-to-DNA ratios and times of transfection (amongst other parameters), we have got an extremely low efficiency of transfection, and the few expressing neurons showed a distribution of βII-spectrin-GreenLantern that did not match our observations of immunolocalization of endogenous βII-spectrin. Taking all these into account, the present version of the manuscript will not include live-cell imaging on expressed tagged βII-spectrin. Given that reviewers found that some statements in the initial submission would have been better supported by live-imaging, we made changes in the manuscript so as to acknowledge the limitations of concluding dynamic mechanisms from fixed samples (see for example last sentences on 5th paragraph of the Discussion section). Having said so, we hope to be able, in the future, to overcome these experimental challenges and be able to establish live-imaging of βII-spectrin in neurons. For example, to avoid unregulated transgene expression, Heller and colleagues recently generated a βII- spectrin-mNeonGreen conditional knock-in (cKI) mice, consisting of a LoxP- flanked alternative final exon of endogenous βII-spectrin with a C- terminal mNeonGreen fusion that is expressed upon Cre expression (Heller et al., 2025). The implementation and further development of such approaches will be very helpful in new studies on the dynamics of βII-spectrin and the MPS as a whole. However, the scale of work needed to accomplish those approaches represent stand-alone projects.

**Reviewer #1 (Recommendations for the authors):**

In the section "The MPS is absent in beta-II spectrin gaps, the authors mention that the presence of MPS in patches suggests that the axons are not undergoing degeneration. I don't think this is a good criterion to use, despite the citations they take support from.

(R17) We agree with the reviewer's suggestion: in virtue of the unlikely connection between the cited developmental axon degeneration process in sensory neurons and the possible axon degeneration of long term cultures of human-iPSCs-derived motor neurons studied here, we have eliminated the sentence of reference

The authors show that degradation by proteases does not happen in their case. In this regard, they may want to discuss the recent article by Heller et al, Science 2025 (https://doi.org/10.1126/science.adn6712) and Hofmann et al, Sci. Rep., 2022 (https://doi.org/10.1038/s41598-022-18562-5)

(R18) By western blot analysis, we did not see evident changes in proteolysis-derived fragments. However it is likely that even when finding phenotypes with protease inhibitors, protein fragments accumulation is below the sensitivity of western blots. We were expecting gross changes observable by western blot in the case proteolysis explained gap formation.

Calpain and Caspase activity has been shown to be relevant in different aspects of MPS biology. To the works cited by the reviewer, now one has to add the very recent work by Fei and colleagues (Fei et al., 2026). We have modified part of the Discussion section to analyse our results in this broader context.

Briefly, Hofmann and colleagues found that acute treatment with calpain inhibitors right before axotomy lead to an increase in percentage of periodic βII-spectrin (referred by authors as “periodicity”) in the regenerated axons in a 2-hour period. Interestingly, the βII-spectrin patches they describe at distal portions did not increase in number, but they increased in size. This indicates that in the particular situation of axonal regeneration calpain activity puts a brake into MPS formation within patches. This invited us to re-examine our own protease inhibition experiments, and measured patch length in this. The new results are shown in Supplementary Fig. 6 and further analysed in the Discussion section. In summary, our changes were much less notable than the ones found in regenerating axons, but follow the same trend: protease inhibitors made patches longer.

On the other hand, Heller and colleagues found in live-imaging studies that calpain activity contributes to the steady-state dynamics of βII-spectrin exchange in a mature MPS lattice. More recently, Fei and colleagues found that caspase or calpain inhibition does not change the steady-state organization of a mature MPS lattice when observing treated axons after fixation samples. Fei and colleagues find a relevant role for calpains whenever massive endocytosis (of any kind) is engaged experimentally. Interestingly, all these studies, including ours, examined calpains roles in MPS in different scenarios. When looked in detail, we don’t believe that these are contradictory results among them, and a complete picture of calpains (and caspases) roles in MPS assembly, growth, maintenance and remodeling will have to take into account all the above mentioned results, including ours. All these analyses are now included in the Discussion section.

Minor comments:

(1) "Recently, it was proposed that this continuous MPS organization arises from the coalescence of discontinuous "patches" of incomplete MPS units that originate in the distal axon and migrate proximally (Zhong et al. 2014)." Please check the citation. Should it be Hoffman et al. 2022?

(R19) The reviewer is correct. The proper citation has now been included.

(2) Is there an established link between ALS and spectrin? I would suggest decreasing the emphasis on this as no clear conclusions are achieved.

(R20) As stated in the text, the study of ALS mutations is justified from two aspects: one aspect is that there are several tubulin and other cytoskeletal proteins whose mutations are linked to ALS (Castellanos-Montiel et al., 2020) and microtubules dynamics has been shown to affect the cortical skeleton (Qu et al., 2017). Second, since human motor neurons are affected in ALS, we thought that a complete characterization of the βII-spectrin cortical cytoskeleton in these cells should include ALS-related mutations. We have now included an a basic MPS description in TDP43 and SOD1 mutation (Suppl. Fig. 5).

The aspect of ALS-related mutations only occupies two short paragraphs in the main text and some panels in Supplementary information. To follow the suggestions by the Reviewer, we have downplayed the relative relevance of these results in the text, without compromising the amount of data we show.

(3) There is a typo in the approximate symbol used for 150 kDa in the section where calpain and caspase activity is reported.

(R21) Typo corrected.

(4) Please add the Latrunculin concentration used in the main text, as it makes it easier for the reader.

(R22) Done.

(5) In the Discussion, paragraph starting with "We further showed ...", there is a typo where Zhong et al is cited.

(R23) Corrected.

(6) Supplementary Figure 1B: attachment instead of 'atachment'.

(R24) Corrected.

(7) Include DIVs or time in the schematic. It is easier for the reader to understand.

(R25) We have now included time references in schematics of Suppl. Fig1B.

(8) Supplementary Figure 1C

Unable to distinguish βII-spectrin and βIII-tubulin in the merged image. Separate figure panels will help.

(R26) The merged images in the reconstructions are merely to better show the tracing individual axons at such low magnification. Relevant portions with only βII-spectrin channels are shown in C1 and C2. Separated individual channels are shown elsewhere across the manuscript.

(9) Supplementary Figure 4D

Why is there so much cleavage product for αII-spectrin across DMSO and treatment? It varied over batches as well. Doesn't this mean that αII-spectrin is going through more proteolytic cleavage? Why?

(R27) The amount of cleavage product for αII-spectrin is not a surprise to us. For instance, although calpains and caspases can potentially process both α- and β-spectrin, in in vivo scenarios where calpain activity is triggered there are much more fragments of α-spectrin being produced (Czogalla & Sikorski, 2005). On the other hand, our staining of cleaved-αII-spectrin by the SNTF antibody by immunofluorescence (Fig4C) parallels the findings by western blot -high levels of cleaved-αII-spectrin across treatments. A similar strong staining using this antibody has been recently shown in the intact axon (Heller et al., 2025). It will be interesting in the future to address if these fragments have any biological significance beyond being mere byproducts of αII-spectrin processing.

**Reviewer #2 (Recommendations for the authors):**

Suggestions for improving the quality of the manuscript:

(1) Live imaging in combination with FRAP assays will help define whether the capture of spectrin from gaps into patches is true. Fixed neurons only provide static information and may not reflect real-time physiological effects.

(R28) See R16 regarding possible live-imaging experiments using tagged βII-spectrin constructs.

(2) Could the presence of F-actin trails in axons facilitate the formation of patches? Will the use of formin/Arp2/3 inhibitors rescue the effect of staurosporine, similar to Latrunculin A?

(R29) Very interesting suggestion. It is likely that different pools of F-actin contribute to the dynamic of MPS formation, and actin trails are definitely worth investigating in this context.

(3) Figure 8 lacks a latrunculin A treated condition? Why is this not present?

(R30) The quantification of that treatment was excluded for space and readability. We have now included the values of group LatA + DMSO in Fig8Cand D and rearranged the whole figure.

(4) Does neuronal stimulation have any effect (KCl treatment) on gaps and patches?

(R31) Very interesting suggestion. Unfortunately, we have not examined whereas neuronal stimulation affects any parameter of the gaps-and-patches structure.

(5) Please check the manuscript for typos and reference insertion points in the text. More than a couple were noted.

(R32) We have corrected typos.

**Reviewer #3 (Recommendations for the authors):**

This is a very interesting study that supports a change in paradigm in the model of MPS lattice formation.

(1) One major concern is the assumption that staudosporine-induced gap and patch formation recapitulates the physiological assembly of gaps and patches of betaII-spectrin, solely based on their morphological similarity. This should be further discussed in the manuscript. Further analysis of additional cytoskeleton components, including microtubules in staurosporine-treated neurons, could also be provided.

(R33) See R14.

(2) In Figure 1E, betaIII-tubulin and NF-H seem to accumulate in betaII-spectrin-rich axonal enlargements. If these are patches, how do you reconcile this finding with Figure 2C-D, where NF-M and alphaII-tubulin are not specifically enriched in betaII-spectrin patches?

(R34) We actually show that axonal enlargements and patches are structurally unrelated, in many aspects. We mention these axonal enlargements as a way to perform an exhaustive characterization of all βII-spectrin features found in these axons.

(3) One technical challenge that limits a more compelling support of the new model of MPS formation is that fixed neurons are imaged, which precludes the observation of patch coalescence. This should be further discussed in the revised version of the manuscript.

(R35) The limitation of the experimental approach is now further discussed (see for example last sentences on 5th paragraph of the Discussion section).

(4) On a more general note, the title of some of the Results sub-sections could be revised to convey the findings of those sub-sections and not the Methods that were used (example: "Quantitave and Qualitative analyses of betII-spectrin distribution....").

(R36) According to the suggestion, we have changed the title of this subsection.

References

Boyer, N. P., Sharma, R., Wiesner, T., Parperis, C., Delamare, A., Pelletier, F., Jullien, N., Bhatt, A. M., Parra-Rivas, L. A., Kearney, P. J., Shavarebi, F., Leterrier, C., & Roy, S. (2026). Spectrin condensates provide a nidus for assembling the axonal membrane-associated periodic skeleton. iScience, 29(1), 114454. https://doi.org/10.1016/j.isci.2025.114454

Castellanos-Montiel, M. J., Chaineau, M., & Durcan, T. M. (2020). The Neglected Genes of ALS: Cytoskeletal Dynamics Impact Synaptic Degeneration in ALS. Frontiers in Cellular Neuroscience, 14, 594975. https://doi.org/10.3389/fncel.2020.594975

Czogalla, A., & Sikorski, A. F. (2005). Spectrin and calpain: A “target” and a “sniper” in the pathology of neuronal cells. Cellular and Molecular Life Sciences: CMLS, 62(17), 1913–1924. https://doi.org/10.1007/s00018-005-5097-0

Guix, F. X., Capitán, A. M., Casadomé-Perales, Á., Palomares-Pérez, I., López Del Castillo, I., Miguel, V., Goedeke, L., Martín, M. G., Lamas, S., Peinado, H., Fernández-Hernando, C., & Dotti, C. G. (2021). Increased exosome secretion in neurons aging in vitro by NPC1-mediated endosomal cholesterol buildup. Life Science Alliance, 4(8), e202101055. https://doi.org/10.26508/lsa.202101055

Heller, E., Kurup, N., & Zhuang, X. (2025). The membrane skeleton is constitutively remodeled in neurons by calcium signaling. Science (New York, N.Y.), 389(6760), eadn6712. https://doi.org/10.1126/science.adn6712

Qu, Y., Hahn, I., Webb, S. E. D., Pearce, S. P., & Prokop, A. (2017). Periodic actin structures in neuronal axons are required to maintain microtubules. Molecular Biology of the Cell, 28(2), 296–308. https://doi.org/10.1091/mbc.E16-10-0727

Zhong, G., He, J., Zhou, R., Lorenzo, D., Babcock, H. P., Bennett, V., & Zhuang, X. (2014). Developmental mechanism of the periodic membrane skeleton in axons. eLife, 3, e04581. https://doi.org/10.7554/eLife.04581

Zhou, R., Han, B., Nowak, R., Lu, Y., Heller, E., Xia, C., Chishti, A. H., Fowler, V. M., & Zhuang, X. (2022). Proteomic and functional analyses of the periodic membrane skeleton in neurons. Nature Communications, 13(1), 3196. https://doi.org/10.1038/s41467-022-30720-x
